# Neurophysiological markers of successful learning in healthy aging

**DOI:** 10.1007/s11357-023-00811-8

**Published:** 2023-05-12

**Authors:** Dawid Strzelczyk, Simon P. Kelly, Nicolas Langer

**Affiliations:** 1 Methods of Plasticity Research, Department of Psychology, University of Zurich, Andreasstrasse 15, CH-8050 Zurich, Switzerland; 2 University Research Priority Program (URPP) Dynamics of Healthy Aging, Zurich, Switzerland; 3 Neuroscience Center Zurich (ZNZ), Zurich, Switzerland; 4 School of Electrical and Electronic Engineering and UCD Centre for Biomedical Engineering, University College Dublin, Dublin, Ireland

**Keywords:** EEG, ERP, P300, Aging, Learning, Memory formation

## Abstract

The capacity to learn and memorize is a key determinant for the quality of life but is known to decline to varying degrees with age. However, neural correlates of memory formation and the critical features that determine the extent to which aging affects learning are still not well understood. By employing a visual sequence learning task, we were able to track the behavioral and neurophysiological markers of gradual learning over several repetitions, which is not possible in traditional approaches that utilize a remember vs. forgotten comparison. On a neurophysiological level, we focused on two learning-related centro-parietal event-related potential (ERP) components: the expectancy-driven P300 and memory-related broader positivity (BP). Our results revealed that although both age groups showed significant learning progress, young individuals learned faster and remembered more stimuli than older participants. Successful learning was directly linked to a decrease of P300 and BP amplitudes. However, young participants showed larger P300 amplitudes with a sharper decrease during the learning, even after correcting for an observed age-related longer P300 latency and increased P300 peak variability. Additionally, the P300 amplitude predicted learning success in both age groups and showed good test–retest reliability. On the other hand, the memory formation processes, reflected by the BP amplitude, revealed a similar level of engagement in both age groups. However, this engagement did not translate into the same learning progress in the older participants. We suggest that the slower and more variable timing of the stimulus identification process reflected in the P300 means that despite the older participants engaging the memory formation process, there is less time for it to translate the categorical stimulus location information into a solidified memory trace. The results highlight the important role of the P300 and BP as a neurophysiological marker of learning and may enable the development of preventive measures for cognitive decline.

**Supplementary Information:**

The online version contains supplementary material available at 10.1007/s11357-023-00811-8.

## Introduction

The aging of our population, with the increasing prevalence of physical and cognitive impairments, poses an increasing challenge to society. Aging is associated with decline in various domains including episodic memory and learning [1–4]. However, there is a substantial variation in the rate, the onset, and the severity of the decline of learning and memory, ranging from mild (i.e., healthy aging) to dramatic cognitive impairments (e.g., Alzheimer’s disease (AD)).

In neuropsychology, learning capacity is mostly assessed by theoretically founded psychological test batteries. These tests have the advantage that the outcome measures typically hold high standards of psychometric properties (i.e., validity and reliability), and that individual outcome measures can be referenced to scores of a norm population. However, the performance measured by psychological tests usually reflects the product rather than processes of successful or impeded learning. As such, psychological tests generally provide little information about the processes of age-related decline in learning [5]. Noninvasive neurophysiological methods, such as electroencephalography (EEG), may overcome these problems by providing insights into the mechanisms of successful or age-related impediments to learning and memory formation processes [6, 7]. EEG is particularly suitable for tracing the dynamics of memory formation processes at the fast pace typical of learning, owing to its high temporal resolution. Event-related potential (ERP) studies have commonly found associations between memory formation and centro-parietal, positive potentials. Two key centro-parietal components are the P300 (here, we refer to the P3b as P300 in the remainder of this article) and broad positivity (BP).

The P300 component is a centro-parietal positivity peaking around 300 ms post-stimulus onset [8]. The P300 is elicited only by task-relevant stimuli and its amplitude decreases as a function of the prior probability of a stimulus, thus providing a neural index of stimulus expectancy [9–19]. In the context of sequence learning, expectancy grows alongside the accumulation of knowledge about the learned material: known stimuli are more expected than unknown stimuli. Although several studies have reported an age-related increase of the P300 latency with a concurrent decrease of its amplitude in adults [3, 7, 20–25], it is not yet known how learning-related modulations of the P300 change with age.

In addition, early memory-related ERP studies reported larger broad centro-parietal positivity (BP) peaking between 300 and 800 ms post-stimulus onset in young individuals for words that were later remembered compared to those later forgotten [16, 26–28]. Latency and amplitude of this component varied depending on the paradigm and stimulus properties [29]. Later, Steinemann et al. [14] demonstrated that the BP amplitude was especially elevated for stimuli being actively committed to memory (i.e., newly learned stimuli).

Previous memory formation research on the P300 and BP has focused mostly on remembered vs. forgotten comparisons and was conducted mainly in young individuals. Consequently, it is largely unknown how aging affects the modulation of P300 or BP amplitudes and latencies over the course of incremental learning. Therefore, the contribution of the present study is to investigate age effects of these neurophysiological measures related to learning. By using a visual sequence learning task, which consists of the repeated presentation of a simple sequence of tokens, we are able to track the progress of gradual memory formation through both neurophysiological and behavioral markers that are precluded in traditional approaches utilizing a remembered vs. forgotten comparison. First, we expect the accuracy and learning rate to be higher in young compared to older individuals. We further predict that these behavioral indices are directly related to neurophysiological characteristics of the ERP components (i.e., P300 and BP amplitudes). More specifically, the sequence knowledge (i.e., accuracy) can relate to correct stimulus expectancy. Hence, we hypothesize that the P300 amplitude will decrease monotonically over the course of learning, as the sequence knowledge strengthens. On the other hand, we predict the BP amplitude, hypothesized here as the signal of active memory trace formation, to be especially elevated at the point where most of the stimuli are actively committed to memory (i.e., highest learning rate). Hence, we expected a positive relationship between BP and learning rate. Overall, we predict similar patterns of neural activation in both age groups but decreased amplitudes and increased latencies in older compared to the young individuals. Finally, we examine whether the learning-related changes in amplitude are able to identify fast and slow learners across the age groups. Such prediction from neurophysiological measures, independent of behavioral indices, could facilitate the development of new diagnostic tools for age-related learning difficulties.

## Methods

### Participants

In the present study, 100 young and 117 older participants were recruited. Data from one young and two older participants were excluded from further analysis due to technical problems (see exclusion criteria below). This resulted in a remaining sample of 99 young (age range 19.7–43.2 years; mean age, 24.97 ± 4.72 years, 39 male, 77 right-handed) and 115 older individuals (58–83.5 years; mean age, 69.1 ± 5.35 years, 55 male, 98 right-handed). Table 1 shows basic demographic details for both age groups. All participants were healthy, reported normal or corrected to normal vision, and no current neurological or psychiatric diagnosis. The young group consisted of graduate students at the University of Zürich or other universities nearby. The older subjects were recruited during lectures within the Senior-University of Zürich. As a compensation, the participants were given course credit or monetary reward (25 CHF/h). This study was conducted according to the principles expressed in the Declaration of Helsinki. The study was approved by the Institutional Review Board of Canton Zurich (BASEC-Nr. 2017–00226). All participants gave their written informed consent before participation.

**Table 1:** Demographics information

	*Young*		*Older*				
	*Mean*	*SD*	*Mean*	*SD*	*t-value*	*df*	*p-value*
Gender	39 m/62f		55 m/64f		*χ*^2^ = 1.29		0.256
Age (years)	24.97	4.72	69.04	5.35	− 63.42	212	8e-140***
Years of education	15.11	3.2	15.39	3.29	− 0.64	210	0.521
MMSE			29.02	1.02			

Note. *SD*, standard deviation; *df*, degrees of freedom; *m*, male; *f*, female; *MMSE*, mini-mental state examination

*p < 0.05. **p < 0.01. ***p < 0.001

### Procedure

The data used in this study was recorded in our laboratory in the context of a larger project. This larger research project aims to quantify age effects on eye movement behavior and EEG recordings of resting-state and other task-based paradigms. In addition, the reliability of behavioral and neurophysiological measures is also assessed. For this reason, the data was collected in two experimental sessions separated by a week. Older participants were given a questionnaire to complete at home regarding basic demographics, handedness, social interactions, and social status. Upon arrival, the older participants performed the mini-mental state exam (MMSE) in order to screen for cognitive impairment and dementia [30]. All participants accomplished a MMSE score above the threshold of 25.

Subsequently, the participants were comfortably seated in a chair in a sound- and electrically shielded Faraday recording cage. The cage was equipped with a chinrest to minimize head movements and a 24-inch monitor (ASUS ROG, Swift PG248Q, display dimensions 531 × 299 mm, resolution 800 × 600 pixels resulting in a display: 400 × 298.9 mm, vertical refresh rate of 100 Hz) on which the experiment was presented. The distance between the chinrest and the monitor was 68 cm.

### Sequence learning task

**Fig. 1:**
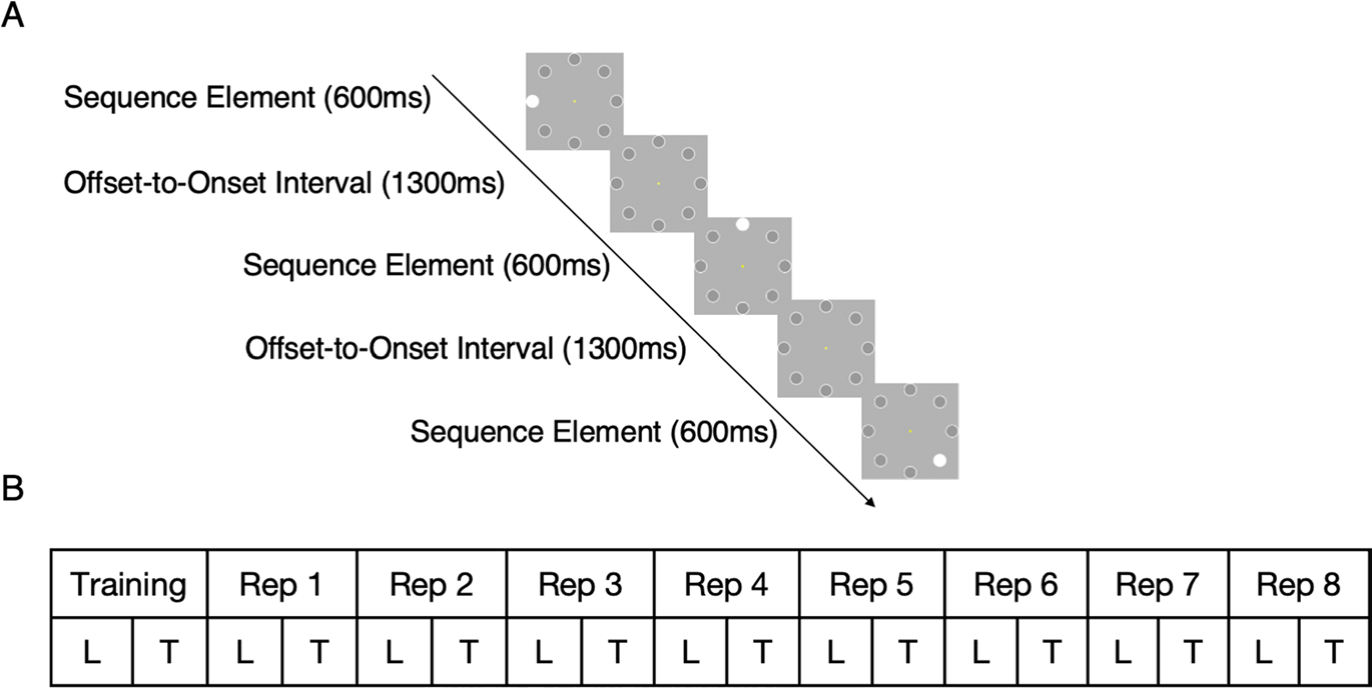
Sequence learning task and the design of the present study. **A** A sequence consisted of eight positions at which white circles were presented one by one. Each stimulus was presented for 600 ms with the interstimulus interval of 1300 ms. **B** Each sequence repetition (Rep) consisted of a learning phase (L) in which all sequence elements were presented and a testing phase (T) in which the participants were asked to recall the position of the stimuli

An explicit visual sequence learning paradigm was first developed by Moisello et al. [31] and is currently considered as an important tool in assessing reliable indices of memory formation and learning progress [14]. The advantage of this paradigm is the simplicity of the stimuli, which enable it to differentiate cortical computations associated with perceptual memorization and stimulus identification [32]. The participants were asked to learn a fixed sequence of eight visual stimulus positions (Fig. 1A). The stimuli consisted of filled white circles (visual angle of 0.84°) and were presented on a computer screen with a bright gray background, positioned equidistant around a ring of fixed eccentricity (visual angle of the distance between center of the screen and the stimulus of 4.21°). Each stimulus was presented for 600 ms with an offset-to-onset interval of 1300 ms. Before the main task recording, a training task was administered, consisting of 4 stimuli placed on the same 8 locations, in order to familiarize the participants with the tasks and to ensure task comprehension. The participants performed the training task until they correctly recalled all 4 locations. Feedback was provided only during the training task.

The main task consisted either of eight sequence repetitions or ended after the participant correctly recalled the sequence of stimuli three times in a row. Each sequence repetition consisted of a learning phase and a testing phase. In the learning phase, the participants were told to focus on a yellow dot at the center of the screen (controlled by an eye-tracking device) and memorize the position of each stimulus. In the test phase, the participants attempted to recall the sequence using a computer mouse by clicking the locations on a computer screen. There was no time restriction on providing responses, and no feedback on their performance was given. The duration of the paradigm for learning a sequence varied between 2 and 5 min, depending on the speed of recall reports and number of sequence repetitions (i.e., 3 to 8 repetitions). Overall, each participant learned six different sequences, resulting in a minimum of 18 (in case the participant solves everything correctly from the beginning) and maximum of 48 sequence repetitions.

### Behavioral data

Each individual stimulus presentation was classified as one of four categories based on the responses of the participants after each sequence repetition. A stimulus was assigned to the *unknown* category (UN) when location was recalled incorrectly in the current and all previous repetitions, to the *newly learned* category (NL) when stimulus position was recalled correctly for the first time in the current repetition, to the *known* category (K) when stimulus position was correctly recalled at least twice in a row, and to the *forgotten* category (F) when stimulus position was once recalled correctly but incorrectly in the following sequence repetition.

To quantify learning performance over sequence repetitions, *accuracy* and *learning rate* were computed for each participant. The *accuracy*, which reflects the cumulative sequence knowledge, was defined as the ratio of the number of correct nCorrect (*P*, *sr*) responses to the total number of stimuli *nT* (*P*, *sr*) in each sequence repetition, where *P* represents the participant and *sr* the sequence repetition.

$$Accuracy\;(P,\;sr)=\frac{nCorrect(P,sr)}{nT(P,sr)}$$

The *learning rate* was defined as the ratio of the number of newly learned stimuli nNL (*P*, *r*) (i.e., stimulus position recalled correctly for the first time) to the total number of stimuli in each sequence repetition.

$$Learning\;rate\;(P,\;sr)=\frac{nNL(P,sr)}{nT(P,sr)}$$

The participants were not instructed to respond as fast as possible; therefore, the reaction time data must be interpreted with caution. For this reason, it was not included in the main manuscript, but in the supplementary material (Supplementary Fig. 3).

#### EEG data acquisition

EEG data were recorded at a sampling rate of 500 Hz using a 128-channel Hydrogel net system (Electrical Geodesics Inc.). The recording reference was at Cz (vertex of the head), and impedances were kept below 40 kΩ.

#### Eye-tracking data acquisition

An infrared video-based eye tracker (EyeLink 1000 Plus, SR Research; http://www.sr-research.com/) recorded eye movements at a sampling rate of 500 Hz and an instrumental spatial resolution of 0.01°. The eye tracker was calibrated and validated before each task with a 9-point grid until the average error for all 9 points was below 1°. In this study, eye tracker data were used in a control analysis accounting for whether the participants maintained focus at the center of the screen as instructed (i.e., yellow dot). Therefore, eye movements during each stimulus presentation period were included into the models as a covariate (i.e., 1 = kept fixation, 0 = lost fixation). It was deemed that a participant had kept fixation, if the gaze was directed at the center of the screen within a square of side 1.26° of visual angle during at least 90% of the total duration of the stimulus presentation period. Importantly, none of the best-fit models included eye movements, indicating no effect on P300 or BP amplitude.

#### EEG data preprocessing

All data were analyzed using MATLAB 2020a (The MathWorks, Inc., Natick, Massachusetts, United States) and RStudio 4.0.2 (R Core Team). The data were preprocessed in Automagic 2.4.3, a MATLAB-based toolbox for automated, reliable, and objective preprocessing of EEG-datasets [33]. In the first step in Automagic, the bad channels were detected using the PREP pipeline [34]. A channel was define as bad based on (1) extreme amplitudes (*z*-score cutoff for robust channel deviation of more than 5), (2) lack of correlation (at least 0.4) with other channels with a window size of 1 s to compute the correlation, (3) lack of predictability by other channels (channel is bad if the prediction falls below the absolute correlation of 0.75 in a fraction of 0.4 windows of a duration of 5 s), and (4) unusual high frequency noise using a *z*-score cutoff for SNR of 5. These channels were removed from the original EEG data. The data was filtered using a high-pass filter with 0.05 Hz cutoff and a low-pass filter with 45 Hz cutoff using the EEGLAB function pop_eegfiltnew [35]. The high-pass filter was chosen based on [36] that recommended a range of 0.01–0.1 Hz for slower cortical potentials such as the P300. Line noise was removed using a ZapLine filter with a passband edge of 50 Hz [37]. Next, independent component analysis (ICA) was performed. However, as the ICA is biased towards high amplitude and low frequency noise (i.e., sweating), the data was temporarily filtered with a high-pass filter of 1 Hz in order to improve the ICA decomposition. Using the pre-trained classifier IClabel [38], each independent component with a probability rating > 0.8 of being an artifact such as muscle activity, heart artifacts, eye activity, line noise, and channel noise were removed from the data. The remaining components were back-projected on the original 0.05 Hz high-pass filtered data. In the next step, the channels identified as bad were interpolated using the spherical interpolation method. Finally, the quality of the data was automatically and objectively assessed in Automagic, thus increasing research reproducibility by having objective measures for data quality. Using a selection of 4 quality measures, the data was classified into three categories: Good (1070 sequences), OK (111 sequences), or Bad (24 sequences). Data was classified as bad, if (1) the proportion of high-amplitude data points (> 30 μV) in the signal is greater than 0.2, or (2) more than 20% of time points show a variance greater than 15 μV across all channels, or (3) 30% of the channels show variance greater than 10 μV, or (4) the ratio of bad channels is greater than 0.3. For further analysis, only the datasets with Good and OK ratings were used. Due to technical difficulties (EEG data not saved properly, missing participant’s responses, MATLAB crash during experiment), data from 45 sequences was not available, resulting in a total of 1205 sequences (i.e., 48 ′276 stimuli).

Subsequent analyses were conducted using EEGLAB [39], an open-source software for processing of electrophysiological signals. First, 23 channels were excluded from further analysis, including 10 EOG channels and 13 channels located on the chin and neck as they capture a little brain activity and are mostly contaminated with muscle artifacts [32, 40, 41]. Next, the data was re-referenced to average reference and segmented from − 100 to 800 ms after stimulus onset (i.e., presentation of white circle on one of the eight positions). The segments were inspected using an amplitude threshold of 90 μV resulting in 6.9% rejected segments on average for each participant.

As for baseline correction, rather than assume no systematic differences between stimulus categories and age groups in the baseline interval, the baseline was taken as a predictor into the linear mixed effect model, allowing the amount of baseline correction to vary across conditions [42]. Otherwise, if there were systemically differences in the baseline interval between conditions, the traditional baseline correction would transfer these differences on later components. This approach has been proven superior outlined in a recent publication [42]. The baseline was computed for each trial as an average of a centro-parietal electrode cluster in a time window of − 100 to 0 ms.

#### P300 and broad positivity extraction

Electrode sites and time windows for computing P300 and BP amplitude were extracted in the following analysis steps: First, to identify the electrode cluster for subsequent statistical analysis, which is representative for all participants, the grand average scalp topographies of unknown and newly learned trials between 200 and 800 ms with a 50 ms step were plotted for young and older participants separately (Supplementary Fig. 1A–B). Only the unknown and newly learned trials were selected because the known trials were not expected to produce a strong P300 peak. Next, the maximal centro-parietal positivity (i.e., P300; ~ 350 ms after stimulus onset) was identified and six electrodes (E54, E55, E61, E62, E78, E79) located around this centro-parietal positivity were chosen for computing the grand average ERP waveforms (Fig. 3A). The scalp topographies for the P300 and BP were highly similar for young and older participants. Thus, the electrode cluster was identical for both age groups.

However, it is well known that the P300 peak latency varies across individuals based on several factors such as age, gender, intelligence, memory capacity, personality, and cognitive impairments including depression and dementia [21, 43, 44]. Furthermore, the intersubject variability of the P300 is somewhat enhanced in older subjects, which might reflect the larger variation of cognitive function in older individuals [45, 46]. The reported age-related increased variability of P300 peak latencies might introduce a bias and underestimate the true P300 amplitude in older individuals, when using a fixed time window for analysis, which is currently the common method in P300 research. For this reason, instead of using fixed time windows for statistical analysis based on grand average ERP, we computed an individual peak and time window for each participant using a method introduced by Liesefeld [47]. The initial search window for individual peaks was defined based on the grand average peaks for each age group individually (P300: 250–490 ms in young, 320–560 ms in older; BP: 500–790 ms in young, 560–790 ms in older). From the Liesfeld [47] approach, we extracted the 50%-area latency, onset, and offset of a component for each participant. The 50%-area latency is the time point, in which a component has reached 50% of its area under the curve. The onset and offset of a component were defined by the time point where the component has reached 60% (and has fallen back below 60%) of its amplitude relative to pre-stimulus baseline (see Liesefeld [47] for details). The 50%-area latency was then used as a measure of components latency and for the computation of the interindividual P300 peak latency variability. The individual onset and offset of a component were used to compute the average P300 and BP amplitudes for each participant’s trial (i.e., average over six centro-parietal electrodes between onset and offset of a component).

### Statistical analysis

Except for the interindividual variability of P300 peaks, for which we computed a Levene’s test [48], we analyzed all behavioral and neurophysiological data using linear mixed effect models. The models were as general as possible at first and were progressively simplified using the lmerTest::step function for linear mixed effect models in R Studio [49] in order to identify the best-fit model. The lmerTest::step function works by iteratively removing one variable from the model and fitting a reduced model. Then, an *F*-test is performed between the full model and the reduced model, and the *p*-value is calculated using Satterthwaite’s approximation. If the *p*-value is below significance level (0.05), the variable is deemed to be important for the model and is retained. If the *p*-value is above the significance level, the variable is deemed to be non-significant and is removed from the model. This process is repeated for each variable in the model until no further variables can be removed [49]. The predictor variables included the repetition number (continuous variable: 1–8), age group (factor of 2 levels: young, older), learning categories (factor of 3 levels: unknown, newly learned, known), baseline (continuous variable), session (factor of 2 levels: 1, 2), eye movements (factor of 2 levels: kept, lost fixation), gender (factor of 2 levels: male, female), stimulus number (factor of 8 levels, i.e., the presentation order of stimuli in a given repetition), subject (factor of 214 levels), and sequence number (factor of 6 levels: 1–6, i.e., each participant attempted to memorize six sequences). The fixed and random effects of the model were specified depending on the goal of the analysis, having in mind that mixed model requires at least 5 levels for a random intercept term to accurately estimate the variance [50]. To overcome the increasing complexity of the models (due to inclusion of baseline), only the variables of interest and their interactions were further interpreted (i.e., repetition number, age group, and learning states) and the covariates were included as controls. Furthermore, we did not examine the interaction effects of covariates such as gender, session, and eye movements, because they are not in the main interest of this paper. The best-fit models and their description are reported in the supplementary material.

#### Behavioral analysis

We began the behavioral analysis by computing for each subject the average number of stimuli and average number of repetitions required to finish the task. Next, we examined the behavioral learning progress of both age groups. Learning performance was formally modeled as the cumulative knowledge about the sequence (i.e., accuracy) and the learning rate in each sequence repetition. We specified two linear mixed effect models with an accuracy and learning rate as dependent variables (continuous variable: 0–1). The fixed effects included the sequence repetition number, age group, interaction of sequence repetition number and age group, session, and gender and the random effects included subject and sequence number. In the following formulas, the sum of model terms is denoted by a “ + ” symbol, the interaction effects by an “ ∗ ” symbol, and the random effects are specified using the vertical bar “(|)” symbol, in line with the Wilkinson notation [51].

$$\mathrm{Accuracy}\;\mathrm{or}\;\mathrm{Learning}\;\mathrm{rate}\sim\mathrm{RepetitionNr}\;\ast\;\mathrm{AgeGroup}+\mathrm{Gender}+\mathrm{Session}+(1\vert\mathrm{Subject})+(1\vert\mathrm{SequenceNr})$$

#### EEG data analysis

##### Interindividual variability of P300 peak latency

To investigate age effects of the variability of interindividual peak latencies, we tested the equality of variances of both age groups by conducting the Levene’s test [48]. Next, we tested whether older participants exhibit delayed P300 peak latencies compared to young participants by fitting a linear mixed model with P300 peak latency as dependent variable, age group as a predictor, and subject as a random effect.

$$\mathrm{Latency}\sim \mathrm{AgeGroup}+(1|\mathrm{Subject})$$

##### P300 and BP amplitude over sequence repetitions

In the next step, assuming the inverse effect of expectancy on P300 amplitude, we investigated the changes in P300 and BP amplitude over sequence repetitions to test the hypothesis that P300 and BP decrease over the course of learning as the expectancy increases, and to assess their eligibility for prediction of learning success. First, we computed average P300 (i.e., mP300) and BP (i.e., mBP) amplitudes in each of the eight sequence repetitions. Subsequently, we defined a linear mixed model for both, average P300 and BP amplitude as dependent variables (continuous variable) and included repetition number, age group, average baseline in a given repetition, interaction of repetition number, age group and baseline, and additionally gender and session as fixed effects and the subject and sequence number as a random effect.

$$\mathrm{mP}300\;\mathrm{or}\;\mathrm{mBP}\sim\mathrm{RepetitionNr}\;\ast\;\mathrm{AgeGroup}\;\ast\;\mathrm{Baseline}+\mathrm{Gender}+\mathrm{Session}+(1\vert\mathrm{Subject})+(1\vert\mathrm{SequenceNr})$$

##### EEG signatures of successful learning

Next, we aimed to investigate whether the P300 and BP amplitudes are directly linked to the behavioral indices and could predict learning success and learning rate over sequence repetitions, respectively. P300 is modulated by stimulus expectancy and in sequence learning expectancy corresponds to increasing knowledge. Hence, we examined the relationship of the average P300 amplitude in each sequence repetition with the accuracy. Meanwhile, the BP, as a signal associated with active memory trace formation, was expected to be especially elevated in repetitions where most stimuli were successfully committed to the memory; hence, we tested the relationship between BP and learning rate. Using linear mixed models, we tested whether the accuracy and learning rate in a given sequence repetition can be predicted by the average P300 and BP in this repetition, respectively. Additionally, we used the age group, average baseline, gender, and session as fixed effects and the subject and sequence number as random effects.

$$\mathrm{Accuracy}\sim \mathrm{mP}300*\mathrm{AgeGroup}*\mathrm{Baseline}+\mathrm{Gender}+\mathrm{Session}+(1|\mathrm{Subject})+(1|\mathrm{SequenceNr})$$

$$\mathrm{Learning}\;\mathrm{rate}\sim\mathrm{mBP}\ast\mathrm{AgeGroup}\ast\mathrm{Baseline}+\mathrm{Gender}+\mathrm{Session}+(1\vert\mathrm{Subject})+(1\vert\mathrm{SequenceNr})$$

##### Predicting learning success across participants

Next, we aimed to predict learning success across participants and investigated if the P300 amplitude decrease could differentiate between slow and fast learners. First, the number of repetitions each participant required to finish the task was obtained (i.e., spanning between 3 and 8 repetitions). Next, the decrease in the P300 amplitude between the first and the third sequence repetition was computed, where most of the learning took place. Finally, we specified a linear mixed effects model with the number of repetitions required to finish the task as the dependent variable (continuous variable). The fixed effects factors included the difference in P300 amplitude between the first and the third sequence repetition (continuous variable), age group, gender, session, and interaction of P300 difference, age group as fixed effects and the subject and sequence number as random effects.

$$\mathrm{Number}\;\mathrm{repetitions}\sim\mathrm P300\;\mathrm{decrease}\ast\mathrm{AgeGroup}+\mathrm{Gender}+\mathrm{Session}+(1\vert\mathrm{Subject})+(1\vert\mathrm{SequenceNr})$$

##### P300 and BP amplitude across learning categories and age groups

The decrease of P300 and BP amplitudes over sequence repetitions might be caused by habituation and occur purely as a function of time spent on the task. To provide stronger evidence for the relationship of both ERP components with learning, we examined the differences in P300 and BP amplitude across learning categories (i.e., unknown, newly learned, and known stimuli). The forgotten category was excluded due to insufficient number of trials (on average 4.7% per participant). For this analysis, we conducted the linear mixed model on single trial data, because the trial-averaged ERP (e.g., averaging across trials within the same condition) has at least two limitations: first, the averaging approach has the drawback of different numbers of stimuli for each participant in each category, which has an impact on the signal-to-noise ratio. Second, it does not take the random variance between subjects and individual differences in effect sizes into account [52, 53]. In order to account for this, we used the linear mixed effect models. The fixed effects included — additionally to the variables of interests (i.e., category and age group) — the baseline, interaction of learning category, age group and baseline, session, eye movements, and gender and the random effects included subject, stimulus number, repetition number, and sequence number.

$$\mathrm P300\;\mathrm{or}\;\mathrm{BP}\sim\mathrm{Category}\ast\mathrm{AgeGroup}\ast\mathrm{Base}+\mathrm{Gender}+\mathrm{Session}+\mathrm{ET}+(1\vert\mathrm{Subject})+(1\vert\mathrm{StimulusNr})+(1\vert\mathrm{SequenceNr})+(1\vert\mathrm{RepetitionNr})$$

However, the linear mixed models do not provide test statistics for all learning states and group comparisons. Therefore, we computed contrasts between the learning states and age groups using the *emmeans* package in R Studio and corrected the *p*-values for multiple comparisons using Bonferroni correction by multiplying *p*-values by the number of comparisons [54]. The adjusted *p*-values smaller than 0.05 (*p* < 0.05) were considered significant. The results of contrast comparisons were averaged over remaining fixed effects.

#### Reliability of behavioral performance and P300 amplitude

High test–retest reliability of outcome measures is essential for future longitudinal studies and is a prerequisite for a biomarker. Therefore, in order to assess the reliability of the performance measures (i.e., number of repetitions required to finish the task) and P300 amplitude within- and between-sessions, we computed the intraclass correlation coefficients (ICCs) for performance and P300. For within-session reliability, we computed the degree of consistency (i.e., norm-referenced reliability; ICC(C, 1) [55]) for performance and P300 across the three sequences in each of two sessions. The P300 for each sequence was defined as the average amplitude over all artifact-free stimuli in the first three sequence repetitions (i.e., 24 stimuli). Sensitivity analysis revealed that the latter approach yielded equivalent results, when defining P300 as a sum over all stimuli in a given repetition. However, this would result in a different number of stimuli per participant; therefore, we opted for the first approach for defining P300 (i.e., the average amplitude over all artifact-free stimuli in the first three sequence repetitions). For between-session reliability (i.e., test–retest reliability), we computed the degree of consistency for measurements that are averages of three measurements (i.e., three sequences per session) of randomly selected participants (ICC(C, k) [55]). First, we computed the average performance and average P300 for each participant in a given session. Performance was defined as the average number of repetitions required to finish the task in a given session and the P300 was defined as the average amplitude over all stimuli in the first three sequence repetitions and over all three sequences in a given session. Based on Cicchetti [56], ICCs less than 0.40 were considered poor, ICC between 0.40 and 0.59 fair, ICC between 0.60 and 0.74 good, and ICC between 0.75 and 1.00 excellent.

## Results

### Behavioral data

#### Demographics

Table 1 shows basic demographic information for young and older participants.

#### Visual sequence learning task performance

Across both sessions (i.e., 6 sequences), young participants required on average 4.56 ± 1.17 repetitions, while older participants 6.57 ± 1.19 repetitions to finish the task, resulting on average in 201.4 ± 10.78 (26.85 ± 5.56 unknown, 49.39 ± 1.59 newly learned, 119.94 ± 4.72 known, 5.22 ± 2.14 forgotten) stimuli per subjects in young and 285.76 ± 13.86 (76.21 ± 9.59 unknown, 59.06 ± 2.99 newly learned, 131.77 ± 7.4 known, 18.73 ± 3.2 forgotten) stimuli per subject in older participants (M ± SD). The task was considered as finished after 8 repetitions or after the participant correctly recalled all sequence elements 3 times in a row (i.e., fastest learners completed the task after 3 and slowest learners after 8 repetitions).

**Fig. 2:**
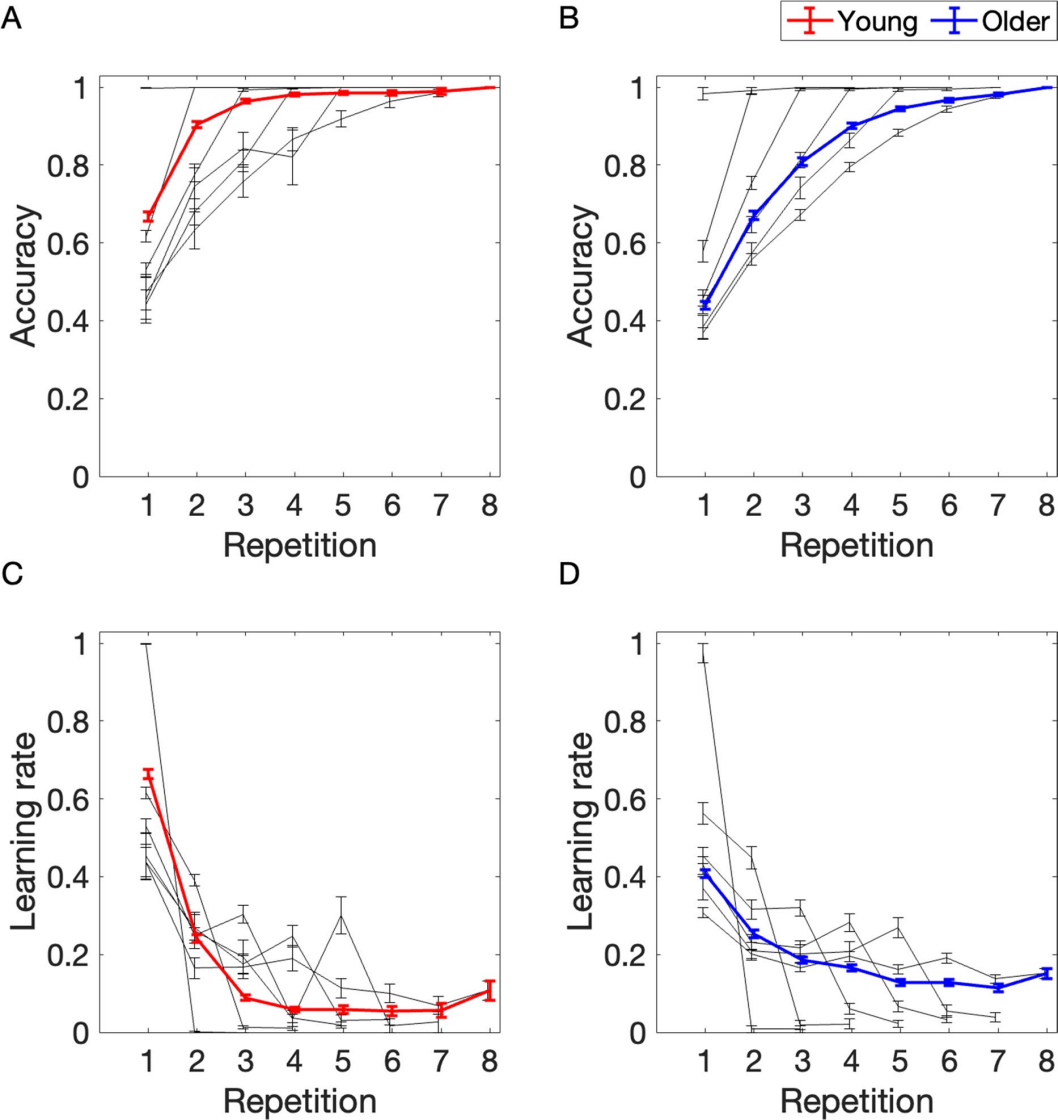
Sequence learning task performance across sequence repetitions in young and older participants. The task lasted 3 to 8 repetitions, depending on learning rate. The black lines indicate the learning curves of participants that completed the task after 3, 4, 5, 6, 7, or 8 sequence repetitions. Error bars represent the standard error of the mean

We next tested the hypothesis that the knowledge about sequence elements increases with each sequence repetition and tested for potential age differences. Figure 2 shows the progression of accuracy and learning rate (see “Behavioral data” section) over the sequence repetition. To visualize the interindividual variance in performance, we further subdivided the participants into groups based on the number of sequence repetitions required for memorization of all sequence elements. The black lines represent the learning curves of each of those groups.

##### Accuracy

The model revealed a significant main effect of repetition number (*β* = 0.07, CI = [0.07; 0.08], *p* = 1.7e-191), that is the accuracy increased with each sequence repetition (Fig. 2A–B). Moreover, there was a significant main effect of age group (*β* =  − 0.21, CI = [− 0.24; − 0.18], *p* = 2.6e-39), suggesting a lower accuracy in the older group. There was also a significant interaction of repetition number and age group, indicating a greater increase in accuracy with each sequence repetition in the older group compared to the increase in the young group (*β* = 0.02, CI = [0.01; 0.02], *p* = 1.9e-5). The young subjects learned most of the sequence elements in the first 2 repetitions, so their increase in accuracy was less pronounced over all repetitions, while the learning of older participants was more evenly spread. In addition, we observed a substantial variation of accuracy between subjects (SD = 0.08) (Supplementary Table 1).

##### Learning rate

There was a significant main effect of repetition number (*β* =  − 0.11, CI = [− 0.12; − 0.11], *p* = 1.5e-303), meaning that the learning rate decreased with each sequence repetition (Fig. 2C–D). The learning rate decreased, because with an increasing number of known stimuli, less stimuli that could be newly learned were available. In addition, there was a significant main effect of the age group, indicating a lower learning rate in the older group (*β* =  − 0.22, CI = [− 0.24; − 0.19], *p* = 7.2e-69) than in the young group. We further observed a significant interaction of repetition number and age group, reflecting the fact that the learning rate decreased less strongly with each sequence repetition in the older group (*β* = 0.07, CI = [0.07; 0.08], *p* = 2.4e-105). Thus, older participants tended to learn fewer sequence items in the first and second repetitions, so that their learning was more evenly spread across repetitions (Supplementary Table 2).

### Neurophysiological data

#### Interindividual variability of P300 peak latency

**Fig. 3:**
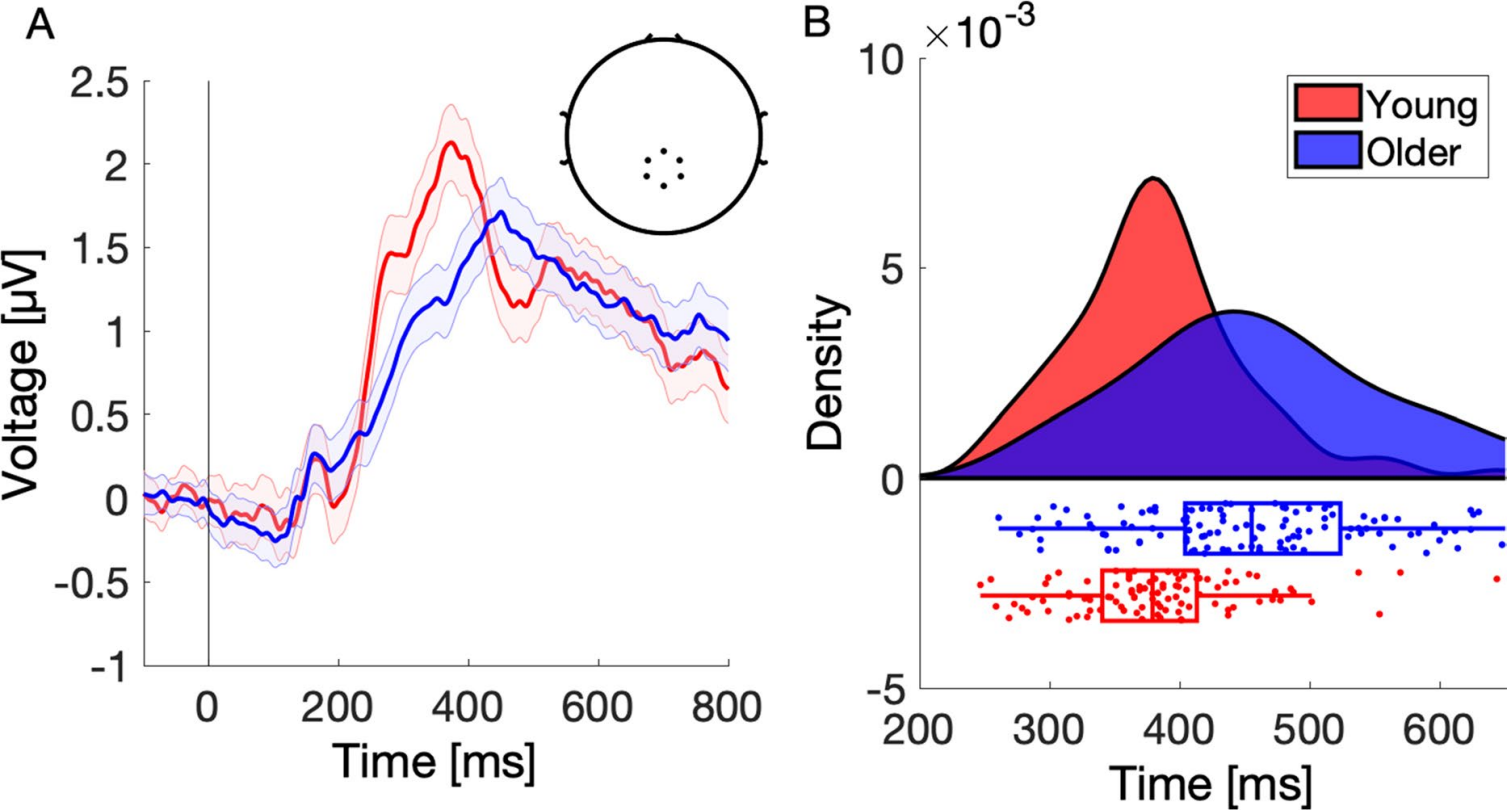
Grand average ERP and interindividual variability of P300 peaks in young (red) and older (blue) participants. **A** Grand average of young and older participants was computed using six centro-parietal channels: E54, E55, E61, E62, E78, E79 (see topoplot). Only the trials from repetitions 1 and 2 were chosen because they comprised the majority of the unknown (i.e., unexpected) stimuli across age groups, resulting in a clear P300 peak. Shaded error bars represent the standard error of the mean. **B** Distribution of individual P300 peaks in young and older participants. Each dot on a boxplot represents a P300 50%-area peak latency

We began the analysis of neurophysiological data by examining the interindividual variability of the P300 peak latency, because the potential amplitude differences between age groups might be overestimated using fixed time windows for extraction of P300. Analyzing the P300 and BP components using the same fixed time window for different age groups might reveal age-related alterations solely driven by latency differences. In addition, if older subjects exhibit an increased variability of P300 peak latencies with advancing age, this might introduce a bias to underestimate the true P300 amplitude in older individuals, even when using a fixed age-adjusted time window, but not accounting for interindividual differences in P300 latency. Therefore, we statistically tested the differences in P300 peak variability using the Levene’s test. The test showed a significant difference between the variances of both age groups (Levene’s (1, 215) = 13.99, *p* = 2e-4). Further inspection of the data revealed greater interindividual variability of P300 peaks in older participants. Next, we confirmed well-established findings that older participants exhibit delayed P300 peak latency compared to young participants (*β* = 78.5, CI = [55.18; 101.82], *p* = 2.2e-10). Summarized, young showed earlier P300 peak latency and smaller P300 peak variability (M = 381.94 ms, SD = 68.44 ms) compared to older participants (M = 460.44 ms, SD = 99.30 ms). To account for the P300 peak latency variability, participant-specific P300 amplitudes were calculated for all subsequent analyses using the Liesefeld (2018) method. Figure 3B shows the distribution of individual P300 peaks in young (red) and older (blue) individuals. These results highlight the importance of computing P300 peaks individually, especially when investigating different age groups.

#### P300 and BP amplitude across sequence repetition

**Fig. 4:**
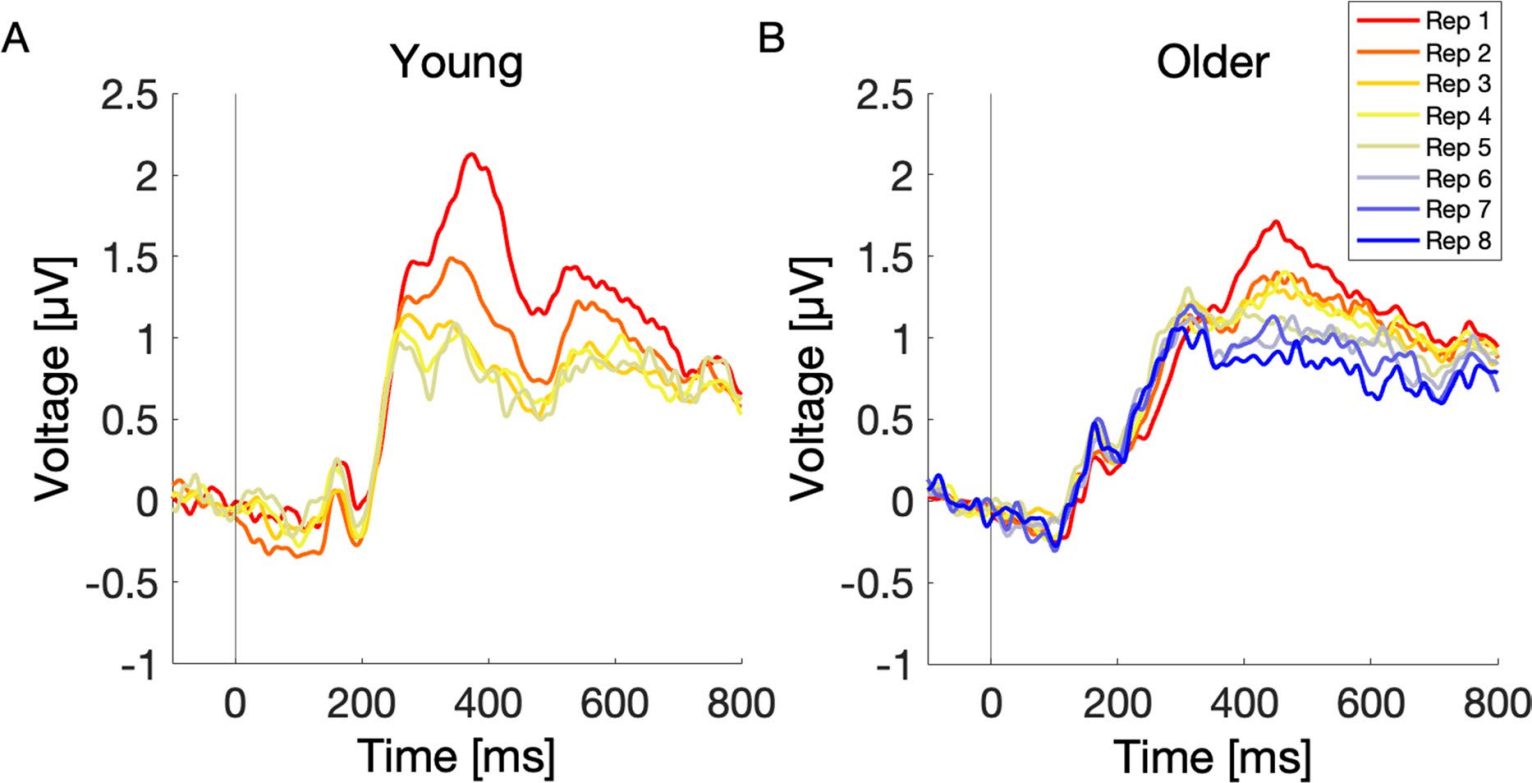
ERPs across sequence repetitions in young (**A**) and older (**B**) individuals. P300 and BP decreased significantly over sequence repetition in both, young and older participants. In young, only the first five sequence repetitions were plotted due to low number of stimuli from the sixth sequence repetition on (only for plotting purposes)

So far, we established on a behavioral level that young and older participants gradually learn the stimuli over repeated sequence presentations, with the young learning on average faster than older participants. In the next step, we tested the hypothesis that the learning progress is accompanied by decreasing P300 (due to increasing expectancy) and BP amplitudes (due to the decreasing need for active memory formation). For that, we computed the average P300 (mP300) and BP (mBP) amplitudes in each sequence repetition (i.e., average P300 and BP amplitude over eight stimuli in a given sequence repetition). Please note, some participants completed the task after just three sequence repetitions, while others needed up to eight. Consequently, the number of overall sequence repetitions decreases after the third repetition. The average ERP waveforms in each sequence repetition are presented on Fig. 4. Panel A shows the ERPs across sequence repetitions in young participants, and panel B shows the ERPs in older participants.

##### P300

The model revealed a significant main effect of repetition number (*β* =  − 0.18, CI = [− 0.21; − 0.15], *p* = 3.7e-41), indicating a decrease of P300 amplitude with each sequence repetition. Furthermore, there was a significant main effect of age group (*β* =  − 0.26, CI = [− 0.41; − 0.10], *p* = 0.001), that is, the P300 amplitude was decreased in older compared to young participants. There was also a significant interaction of repetition number and age group (*β* = 0.11, CI = [0.08; 0.14], *p* = 8.6e-14), indicating a smaller decrease of P300 amplitude across sequence repetitions in older participants (Table 2).

**Table 2:** Effects of repetition number and age group on P300 amplitude

*Variable*	*β*	*SE*	*CI*	*t-value*	*p-value*
Intercept	1.37	0.07	1.24–1.51	21.01	8.6e-51***
RepetitionNr	− 0.18	0.01	− 0.21 to − 0.15	− 13.53	3.7e-41***
AgeGroup	− 0.26	0.08	− 0.40 to − 0.10	− 3.24	0.001**
RepetitionNr*AgeGroup	0.11	0.02	0.08–0.14	7.47	8.6e-14***
Variance components	SD	Goodness of fit			
Subject	0.42	Log likelihood		− 9292.25	
SequenceNr	0.05				
Residual	0.99				

Note. Intercept represents the first repetition of young participants. *RepetitionNr*, repetition number; *SequenceNr*, sequence number; *β*, unstandardized regression coefficient; *SE*, standard error; *CI*, confidence interval; *SD*, standard deviation

*p < 0.05. **p < 0.01. ***p < 0.001

##### Broad positivity

This model revealed a significant main effect of repetition number (*β* =  − 0.11, CI = [− 0.14; − 0.08], *p* = 5e-16), indicating a decrease in BP amplitude with each sequence repetition. The main effect of age group (*β* =  − 0.13, CI = [− 0.27; 0.01], *p* = 0.054) was not statistically significant, providing not enough evidence for age-related differences in overall BP amplitude aggregated across repetitions between young and older participants. However, there was a significant interaction of repetition number and age group (*β* = 0.06, CI = [0.01; 0.08], *p* = 3.3e-4), reflecting a more gradual decrease of BP amplitude across sequence repetitions in older participants (Table 3).

**Table 3:** Effects of repetition number and age group on BP amplitude

*Variable*	*β*	*SE*	*CI*	*t-value*	*p-value*
Intercept	0.89	0.07	0.78–1.01	14.69	1.2e-41***
RepetitionNr	− 0.11	0.01	− 0.14 to − 0.08	− 8.24	5e-16***
AgeGroup	− 0.13	0.08	− 0.27–0.01	− 1.93	0.054
RepetitionNr*AgeGroup	0.06	0.02	0.01–0.08	3.59	3.3e-4***
Variance components	SD	Goodness of fit			
Subject	0.30	Log likelihood		− 9440.81	
Residual	1.06				

Note. Intercept represents the first repetition of young participants. *RepetitionNr*, repetition number; *β*, unstandardized regression coefficient; *SE*, standard error; *CI*, confidence interval; *SD*, standard deviation

*p < 0.05. **p < 0.01. ***p < 0.001

#### EEG signatures of successful learning

Having established that the P300 and BP decrease with sequence repetition, we examined the potential of the P300 and BP in predicting learning success across sequence repetitions, measured as accuracy and learning rate. For that, we computed the average P300, BP, and baseline amplitude in each sequence repetition for each participant.

##### Accuracy and P300

Expectancy-driven P300 was hypothesized to decrease, as the sequence knowledge strengthened. Thus, we tested whether the P300 amplitude could predict the accuracy across sequence repetitions. The model revealed a significant main effect of P300 (*β* =  − 0.04, CI = [− 0.04; − 0.03], *p* = 2.2e-36) that is the accuracy increased with decreasing P300 amplitude. Furthermore, there was a significant main effect of age group (*β* =  − 0.09, CI = [− 0.10; − 0.07], *p* = 2.5e-23), that is, the accuracy was lower in older compared to young participants (Supplementary Table 5).

##### Learning rate and broad positivity

BP, the signal thought to reflect active memory trace formation, was expected to be maximal for stimuli being actively committed to memory. Thus, we tested whether the learning rate, which represents the proportion of newly learned stimuli in a given repetition, could be predicted by the average BP amplitude across sequence repetitions. The model revealed a significant main effect of the BP (*β* = 0.05, CI = [0.04; 0.06], *p* = 3.1e-25), indicating a positive relationship between the learning rate and BP. The main effect of the age group did not reach significance (*β* = 0.00, CI = [− 0.01; 0.02], *p* = 0.621), but a significant interaction of age group and BP (*β* =  − 0.04, CI = [− 0.05; − 0.03], *p* = 4.5e-10) indicated that learning rate varied less sensitively with BP amplitude in the older group (Supplementary Table 6).

#### Predicting learning success across participants

**Fig. 5:**
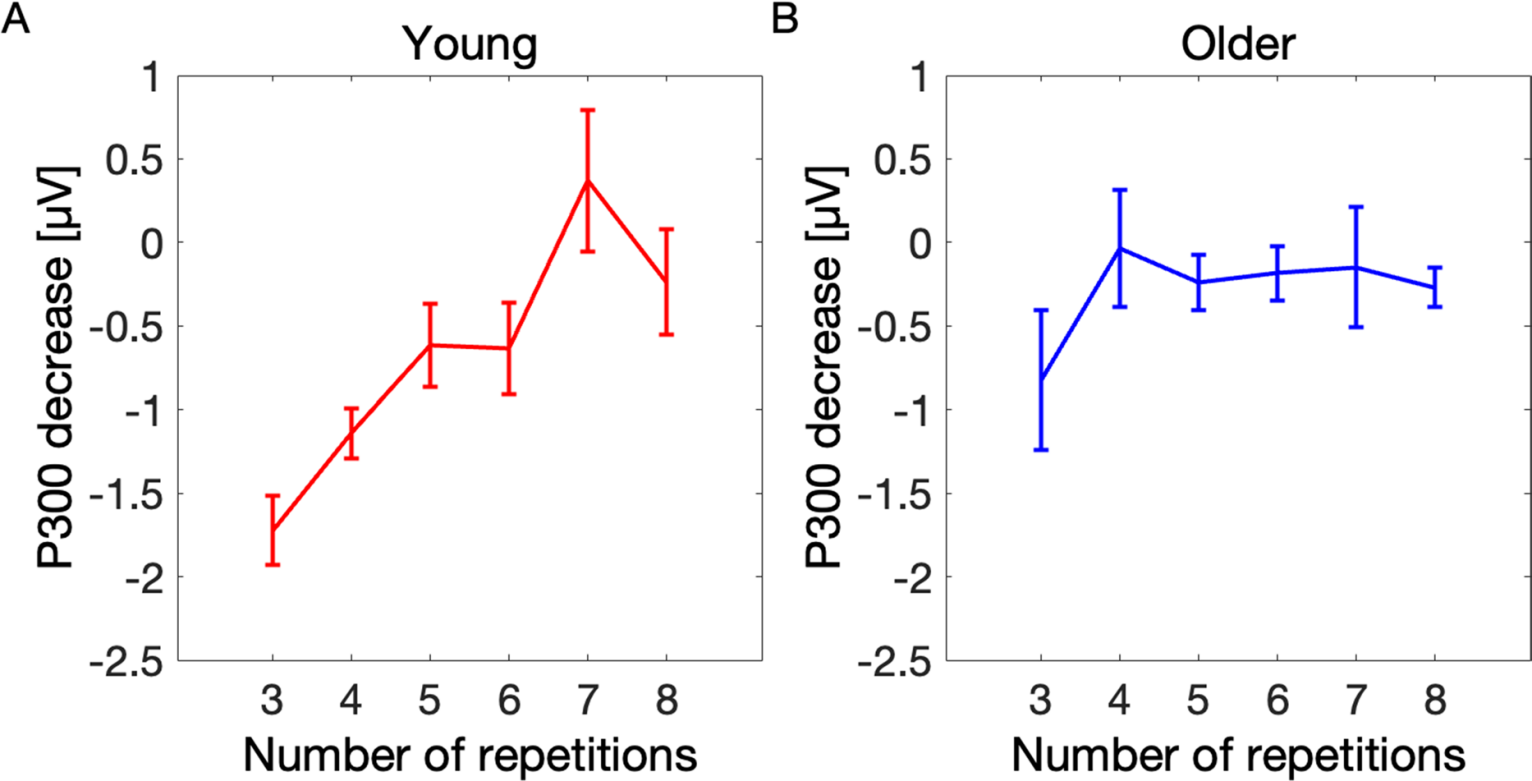
The decrease of P300 amplitude during learning could identify fast and slow learners in young, but not in older participants. Young participants that finished the task already after 3 repetitions showed the greatest P300 amplitude difference between the first and third sequence repetitions, and the amplitude difference was decreasing with increasing number of repetitions required to finish the task. The effect was weaker in older participants. The error bar represents the standard error of the mean

In the next step, we aimed to predict the learning performance across participants (i.e., to investigate if the P300 amplitude decrease can differentiate between slow and fast learners) based on the decrease of the P300 amplitude between the first and third sequence repetitions (Fig. 5). Learning performance was defined as the number of sequence repetitions required to finish the task (i.e., numReps). The model revealed a significant main effect of P300 decrease (*β* = 0.08, CI = [0.03; 0.12], *p* = 0.001) indicating more repetitions (i.e., slower learning) with smaller P300 decrease. Moreover, the significant main effect of age group (*β* = 1.91, CI = [1.63; 2.19], *p* = 4e-30) showed higher number of repetitions in older compared to that of young participants, substantiating the findings of behavioral analysis that the older learned significantly slower than the young individuals. There was also a significant interaction of P300 decrease and age group (*β* =  − 0.08, CI = [− 0.15; − 0.01], *p* = 0.022), indicating that for the older participants, the P300 decrease was less predictive of the number of repetitions required to finish the task (Supplementary Table 7).

#### P300 and BP amplitude across learning categories and age groups

**Fig. 6:**
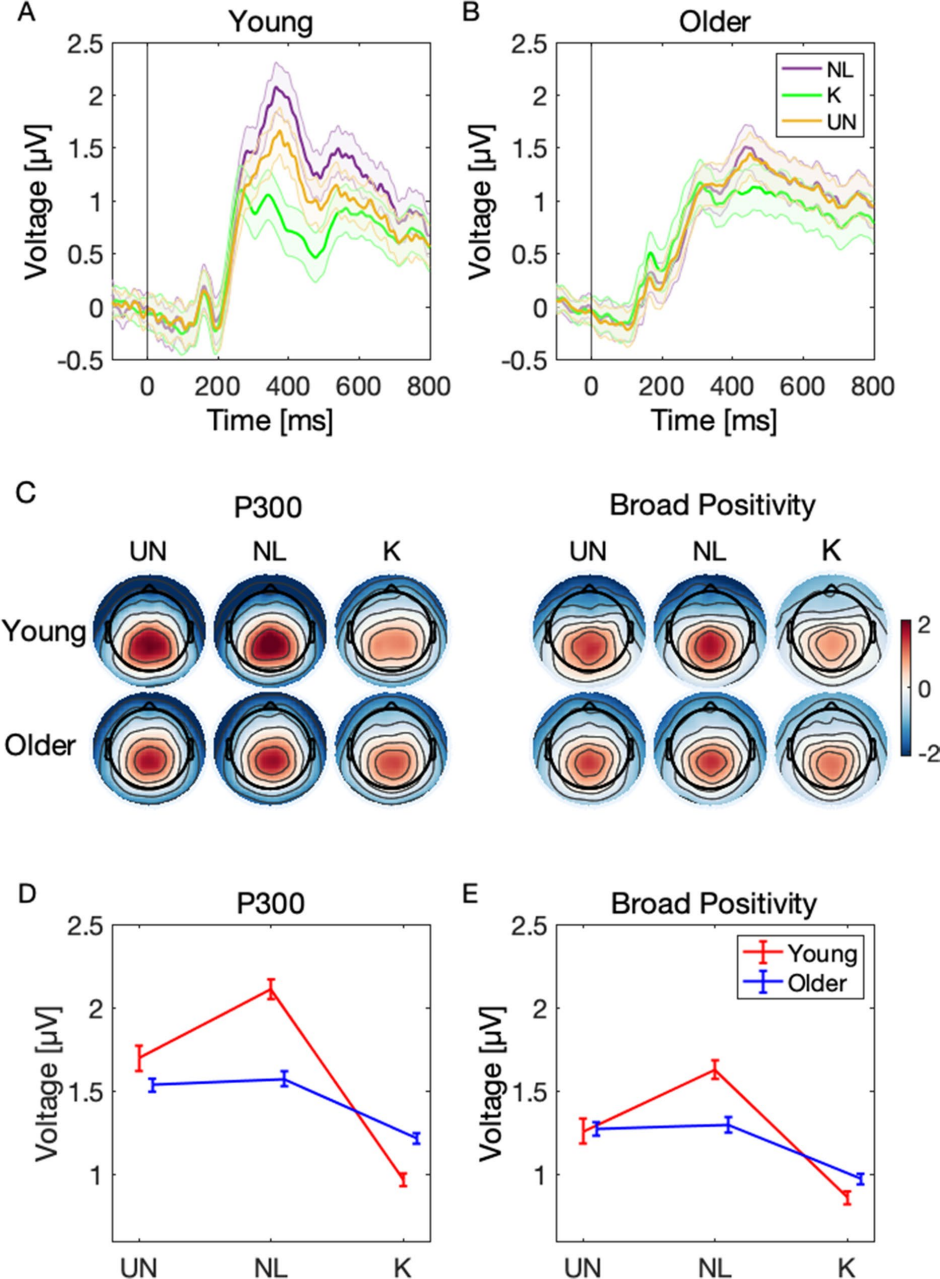
Event-related potentials, topographical maps, and mean amplitudes over learning categories computed based on individual peaks. **A**–**B** Centro-parietal ERPs of young (left) and older (right) participants across learning categories: unknown, newly learned, and known. Shaded error bars represent the standard error of the mean. **C** Scalp topographies of the P300 and broad positivity for each learning state and age group. **D**–**E** The results of contrast comparisons revealed that the P300 amplitudes were greater for unknown and newly learned trials than for known trials in both age groups; however, the amplitude difference between unknown and newly learned trials did not reach significance. Moreover, the P300 amplitude of newly learned trials was greater in young compared to older participants, and the P300 amplitude of known trials was significantly greater in older compared to young participants. BP showed the same effects during learning as the P300 within both age groups (UN = NL > K); however, no age differences between contrasts were found. The error bar represents the standard error of the mean

So far, we have demonstrated that the P300 and BP amplitudes decrease with sequence repetitions. Furthermore, our findings suggest that the P300 amplitude can predict behavioral accuracy, and that the behavioral learning rate across sequence repetition can be predicted by the BP amplitude. However, the decrease of amplitude could be simply an effect of habituation and the component’s amplitude could decrease as a function of time spent on a task. Therefore, to provide stronger evidence for the relationship of P300 and BP with learning, we tested whether the amplitude of both components changes as a function of learning state, that is, from stimulus being unknown to newly learned and finally fully known, regardless of the sequence repetition number. First, we identified the best-fit model for the P300 and BP amplitude (Supplementary material). Because we were merely interested in amplitude differences across learning categories and age groups, we additionally computed contrasts for those variables using the emmeans package in R Studio. Figure 6A–C show the ERP waveforms and scalp topographies of young and older participants averaged over six centro-parietal electrodes as a function of the learning category. Figure 6D–E display the P300 and BP amplitudes across learning categories in young and older participants corrected for interindividual P300 peak latency variability.

##### P300

The model revealed a significant main effect of category K (*β* =  − 0.62, CI = [− 0.72; − 0.53], *p* = 2.6e-38), indicating decreased P300 amplitude for K compared to NL (i.e., intercept) trials but a significant interaction of category K and age group (*β* = 0.43, CI = [0.32; 0.55], *p* = 8.5e-14) indicated that P300 amplitude of category K decreased less in the older group. Moreover, there was a significant main effect of age group (*β* =  − 0.21, CI = [− 0.36; − 0.06], *p* = 0.006), suggesting lower P300 amplitude in older participants. No other effects and interaction effects reached significance (Supplementary Table 8). Next, we proceeded with computing contrasts between categories and age groups. In young participants, the results of contrast comparisons showed a significantly greater P300 amplitude for UN compared to K (*β* = 0.49, CI = [0.37; 0.61], *p* = 5e-16) trials. Moreover, the P300 amplitude was significantly larger for NL than K (*β* = 0.65, CI = [0.57; 0.75], *p* = 3.3e-43) trials. The difference between NL and UN trials was not statistically significant (*β* = 0.17, CI = [0.04; 0.28], *p* = 0.053). Similarly, in older participants, the P300 amplitude was significantly greater in UN than K (*β* = 0.13, CI = [0.05; 0.21], *p* = 0.009) and NL than K (*β* = 0.20, CI = [0.12; 0.28], *p* = 8.1e-06) trials. The amplitude differences between NL and UN (*β* = 0.06, CI = [− 0.02; 0.14], *p* = 0.999) did not reach significance. Finally, we compared the P300 amplitude in each learning category between age groups: there were no significant differences between age groups for the UN trials (*β* = 0.16, CI = [0.00; 0.32], *p* = 0.415), but young participants had significantly greater P300 amplitude for the NL trials (*β* = 0.26, CI = [0.12; 0.41], *p* = 0.004) and significantly smaller amplitude for the K trials (*β* =  − 0.20, CI = [− 0.33; − 0.07], *p* = 0.026). The results of contrast comparisons are summarized in Table 4 and visualized in Fig. 6D.

**Table 4:** P300: contrast comparisons between age groups and learning categories

Contrast	*β*	*SE*	*CI*	*z-ratio*	*p-value*
UN Young—K Young	0.49	0.06	0.37–0.61	7.95	5e-16***
NL Young—K Young	0.65	0.05	0.57–0.75	13.94	3.3e-43***
NL Young—UN Young	0.17	0.06	0.04–0.28	2.77	0.053
UN Old—K Old	0.13	0.04	0.05–0.21	3.30	0.009**
NL Old—K Old	0.20	0.04	0.12–0.28	4.91	8.1e-06***
NL Old—UN Old	0.06	0.04	− 0.02–0.14	1.53	0.999
UN Young—UN Old	0.16	0.08	0.00–0.32	1.99	0.415
NL Young—NL Old	0.26	0.08	0.12–0.41	3.52	0.004**
K Young—K Old	− 0.20	0.07	− 0.33 to − 0.07	− 2.98	0.026*

Note. *UN*, unknown; *NL*, newly learned; *K*, known; *β*, unstandardized regression coefficient; *SE*, standard error; *CI*, confidence interval. *p*-values adjusted using the Bonferroni method for 9 tests

*p < 0.05. **p < 0.01. ***p < 0.001

##### Broad positivity

The broader centro-parietal positivity was also investigated with regard to the learning states. The model revealed a significant main effect of category K (*β* =  − 0.43, CI = [− 0.55; − 0.33], *p* = 2e-17), indicating decreased BP amplitude for K compared to NL (i.e., intercept) trials but a significant interaction of category K and age group (*β* = 0.16, CI = [0.04; 0.29], *p* = 0.008) indicated that BP amplitude of category K decreased less in the older group. Further, there was a significant main effect of age group (*β* =  − 0.15, CI = [− 0.28; − 0.02], *p* = 0.021), suggesting lower BP amplitude in older participants (Supplementary Table 9). Next, we proceeded with computing contrasts between categories and age groups. In young participants, the contrast comparison revealed a significantly larger BP amplitude for UN relative to K (*β* = 0.30, CI = [0.18; 0.42], *p* = 9e-6) and NL relative to K (*β* = 0.42, CI = [0.33; 0.52], *p* = 3.7e-17) trials. The BP amplitude of NL and UN trails was not statistically different (*β* = 0.12, CI = [0.00; 0.25], *p* = 0.451). Similarly, in older participants, we observed a larger amplitude for UN than K (*β* = 0.27, CI = [0.19; 0.35], *p* = 4e-10) and NL than K (*β* = 0.28, CI = [0.19; 0.36], *p* = 1.5e-10) trials, while the difference between UN and NL trials did not reach significance (*β* = 0.01, CI = [− 0.07; 0.10], *p* = 0.999). When comparing both age groups, we found no differences in the UN (*β* = 0.04, CI = [− 0.07; 0.10], *p* = 0.999), NL (*β* = 0.16, CI = [− 0.03; 0.29], *p* = 0.128), and K (*β* = 0.01, CI = [− 0.09; 0.11], *p* = 0.999) trials. The results of contrast comparisons are summarized in Table 5 and visualized in Fig. 6E.

**Table 5:** Broad positivity: contrast comparisons between age groups and learning categories

Contrast	*β*	*SE*	*CI*	*z-ratio*	*p-value*
UN Young—K Young	0.30	0.06	0.18–0.42	4.89	9e-6***
NL Young—K Young	0.42	0.05	0.33–0.52	8.66	3.7e-17***
NL Young—UN Young	0.12	0.06	0.00–0.25	1.96	0.451
UN Old—K Old	0.27	0.04	0.19–0.35	6.56	4e-10***
NL Old—K Old	0.28	0.04	0.19–0.36	6.74	1.5e-10***
NL Old—UN Old	0.01	0.04	− 0.07–0.10	0.23	0.999
UN Young—UN Old	0.04	0.07	− 0.09–0.19	0.54	0.999
NL Young—NL Old	0.16	0.07	− 0.03–0.29	2.45	0.128
K Young—K Old	0.01	0.05	− 0.09–0.11	0.27	0.999

Note. *UN*, unknown; *NL*, newly learned; *K*, known; *β*, unstandardized regression coefficient; *SE*, standard error; *CI*, confidence interval. Results are averaged over the levels of session. *p*-values adjusted using the Bonferroni method for 9 tests

*p < 0.05. **p < 0.01. ***p < 0.001

### Reliability of behavioral performance and P300 amplitude

Finally, we computed the reliability of behavioral and electrophysiological measures related to learning to provide information on their within- and between-sessions stability. Behavioral performance was defined as the number of sequence repetitions required to memorize the whole sequence (i.e., 3 to 8). For within-session reliability, the P300 for each sequence was defined as the average amplitude over all artifact-free stimuli in the first three sequence repetitions, resulting in an average of 22.67 ± 2.97 (M ± SD) stimuli per sequence. For test–retest reliability, the P300 for each session was defined as the average amplitude over all stimuli in the first three sequence repetitions and over all three sequences in a given session, resulting in an average of 65.81 ± 10.56 (M ± SD) artifact-free stimuli per session. Within the first session, we found excellent reliability for the performance (ICC = 0.75) and good reliability for P300 amplitude (ICC = 0.63). Within the second session, we found good reliability for the performance (ICC = 0.63) and poor reliability for P300 amplitude (ICC = 0.38). Finally, performance showed excellent (ICC = 0.81) and P300 amplitude good (ICC = 0.71) test–retest reliability across 1 week (i.e., two sessions). Sensitivity analysis revealed that the ICCs were robust across both age groups. Our results demonstrate that the number of repetitions required to finish the task provides fair to excellent reliability and P300 amplitude provides poor to good reliability to measure learning performance and neurophysiological processes related to learning and memory formation.

## Discussion

In the present study, we investigated the effects of aging on learning trajectories and neural processes that contribute to memory formation. The employed visual sequence learning paradigm, in which a fixed sequence of spatially distinct stimuli was memorized over repeated observations, enabled us to track the gradual nature of learning that is precluded in traditional approaches utilizing a remembered vs. forgotten comparison. Our results show that the less effective sequence memorization evident in the behavioral reports of older participants is systematically reflected in a more gradual decrease of neural activity components across repetitions. These neural activity components reflect the degree of expectancy (i.e., P300) and the process of new memory formation (i.e., BP). The findings of our study suggest that these neurophysiological markers have the potential to be a useful tool for monitoring learning effectiveness, regardless of individual’s behavioral reporting abilities. The results are substantiated by a good P300 test–retest reliability. In the following, we incorporate the findings into the current theories about centro-parietal ERP components and memory formation.

### The role of centro-parietal ERP activity during memory formation

Consistent with previous research, we demonstrated lower learning performance in older participants defined in this study as accuracy and learning rate [3, 4, 20, 22, 46, 57]. Lower performance of older participants may be linked to previously reported slowing processing speed, which might be caused by decreased axon myelination and reduction in neurotransmitter levels [58, 59]. Further, we showed that the P300 peak latencies vary between participants, and that the P300 peak variability was increased in older participants. P300 peak variability may be influenced by several factors such as age and memory capacity [21, 43, 44]; therefore, the longer P300 peak latencies in older participants may suggest that they require more time on stimulus categorization and evaluation [60]. These findings substantiate that by employing a fixed time window for analysis, which is currently the standard approach in ERP research, the documented age-related higher variability of P300 peak latencies can create a bias and underestimate the actual P300 amplitude in older individuals.

A core principle in this study was that P300 amplitude decreases as a function of subjective stimulus expectancy [8–13, 16–19, 25, 61], which in the context of sequence learning corresponds to knowledge about the sequence. However, a recent study advocated for an attentional role of the P300, such that it should increase for stimuli as they are better stored in memory, which is opposite to the expectancy/surprise account [62]. We found that P300 decreased over the course of learning, which is in line with the decremental surprise linked to increasingly confident sequence knowledge. The gradual decrease of amplitude over the course of several repetitions after committing the stimuli to memory might reflect the increasing confidence about the sequence, as correct recall might not be synonymous with having no doubts.

Previous ERP research on memory revealed increased broad centro-parietal positivity (i.e., broad positivity) approximately 300–800 ms after stimulus for items later being remembered compared to those forgotten in various tasks [16–19, 27, 28, 61, 63–65]. This “difference in subsequent memory” effect has been observed in a wide range of encoding conditions, although some studies failed to find this effect (for a review, see Johnson [29]). However, others argued that the broad centro-parietal amplitude is not primarily associated with the memorization itself, but rather mediated by stimulus complexity and semantic processing depth [66]. Using simple visual stimuli with minimal semantic value as in the present sequence learning task, where there are no differences in complexity across items, we provide evidence that these modulations of centro-parietal ERP activity are related to memory formation. Although stimuli of low semantic content have been used previously, the majority of these studies analyzed the neurophysiological signature during the recognition rather than encoding [65, 67–69]. As the recognition process is inseparably compounded with decision processes to determine if the stimulus was seen before [67, 68], the exact role of the BP during encoding remained unclear. Moreover, these traditional memory examination methods had the limitation that their design did not allow to track the gradual memory formation processes. We addressed this specifically in our study by utilizing a paradigm which naturally provides the opportunity to investigate the incremental nature of learning. Therefore, we were able not only to compare the P300/BP amplitude of remembered and not remembered stimuli but also examine how the amplitude changes during learning over multiple repetitions. In line with the increasing expectancy associated with increasing sequence knowledge, the P300 declined monotonically over the course of learning and the BP was especially elevated for the repetitions with the highest learning rate. Moreover, our data demonstrate that the slower memorization in older participants was directly linked to more gradual modulations of the P300 and BP amplitude over the course of learning reflecting more spread-out learning progress.

The relationship between the modulations of P300 and BP over the course of learning might also be explained by mere habituation processes [70, 71]. Therefore, we aimed to provide a stronger test for the relationship between neural signals and memory formation by examining amplitude modulations independently of the sequence repetition number. For this reason, we further subdivided the stimuli into different learning states: unknown, newly learned, and known to investigate in more detail the changes in P300 and BP over the course of learning. An additional motivation for the distinction of these different learning states is the fact that the first repetition had the lowest accuracy and the highest learning rate. As the number of repetitions increased, accuracy improved and learning rate decreased monotonically. This opposite, but similar trends made it difficult to disentangle the distinct functions theorized for P300 and BP, as P300 decreased with increasing accuracy and BP decreased with decreasing learning rate.

Consistently across age groups, contrast comparisons demonstrated that both components showed significantly greater amplitude for unknown and newly learned trials compared to known trials. The difference between unknown and newly learned trials did not reach significance. When comparing both age groups, young had significantly greater P300 amplitude for newly learned and significantly smaller amplitude for known trials than older participants. Interestingly, we found no age-related difference in P300 amplitude of unknown trials. Although when examining the P300 amplitude over sequence repetitions, young exhibit greater amplitude in the first repetition and a steeper monotonic decrease afterwards. The fact that unknown P300 amplitudes were not elevated in the young participants despite elevated P300s in repetition 1 might be explained by the fact that a large portion of the young participants learned the majority of the sequence elements already during the first sequence repetition. The unknown category therefore consists of the subset of trials and young participants for which learning was slower, and to the extent that this was linked with lower attentional resources, it may be associated with relatively smaller P300 amplitudes [21]. Indeed, the slower learning rate in older participants might be in some part attributable to the relatively small amplitude and long latency of the P300 peak for newly learned trials, as less efficient stimulus identification likely hinders better memorization of those trials. Further, the drop of the P300 amplitude after committing the stimulus to memory (i.e., from newly learned to known) was steeper in young than in older participants, as shown by the significant interaction of age group and categories newly learned (i.e., intercept) and known (Supplementary Table 8). Moreover, young exhibited smaller P300 amplitudes of the known trials compared to known trials of older participants. These findings confirm previous interpretations that P300 reflects the construct of uncertainty and may suggest that in this undemanding task, young people exhibit high levels of confidence in their knowledge shortly after committing the stimulus to memory, whereas older participants still express some level of doubt.

In addition, one obvious prediction could have been that older people are less efficient learners because their memory formation activity is weaker. However, contrast comparisons showed no age differences in BP amplitude across all learning categories. The lack of appreciable reduction in BP amplitude in the old compared to young is remarkable. It seems that despite the memory formation process being engaged to a similar strength, this engagement does not translate into the same learning progress in the older participants. One possibility is that the slower and more variable timing of the stimulus identification process reflected in the P300 latency means that despite the older participants engaging the memory formation process, there is less time for it to translate the categorical stimulus location information into a solidified memory trace. Nonetheless, the fact that the BP is less effective at generating strong memory traces is borne out in our analysis showing lower beta weights linking the BP to learning rate in the older participants.

### Predicting learning success

So far, we have provided evidence for the contribution of the P300 and BP in sequence learning and further demonstrated neurophysiological foundations of age-related differences in learning observed in behavioral reports. Subsequently, we investigated if these components were effective in predicting learning success within and across participants and potentially could serve as a biomarker for successful learning. To qualify as a biomarker, a prerequisite is a sufficient within-subject reliability of these neurophysiological and behavioral measures. Critically, our results demonstrated excellent test–retest reliability for the behavioral performance and good test–retest reliability for the P300 amplitude. The analysis of within-session reliability revealed good reliability within the first session, but poor reliability within the second session. This inconsistency could be attributed to the insufficient number of trials utilized for the within-session reliability analysis. Previous research conducted by Thigpen et al. [72] demonstrated that increasing the number of trials from 20 to 70 substantially enhanced the signal-to-noise ratio and reliability of P300. Therefore, it is essential to use an adequate number of trials in the reliability analysis to obtain accurate and reliable results, and the within-session ICCs reported here should be interpreted with caution. In contrast, for the test–retest reliability analysis, we pooled the stimuli within the first and second sessions, resulting in an average of 65.81 ± 10.56 (M ± SD) stimuli per session and leading to more reliable results.

Next, we examined whether P300 and broad positivity were predictive for the behavioral measures defined as the accuracy and learning rate. In both age groups, the results revealed that the average P300 amplitude in a given repetition could predict the cumulative knowledge about the sequence (i.e., accuracy) within that repetition. In addition, there was a positive association between broad positivity and learning rate. Finally, we aimed to identify fast and slow learners solely based on the decrease of P300 amplitude during learning. We found that the decrease in P300 amplitude between the first and third sequence repetitions was significantly related to learning performance, as measured by the number of sequence repetitions required to complete the task. Specifically, the fastest learners showed greatest reduction in P300 amplitude and the worse the learning performance the smaller P300 decrease. These findings suggest that the P300 component may be a useful marker of learning and memory, and that it may be sensitive to individual differences in cognitive function. Predictions of learning success from neurophysiological measures, independent of behavioral indices, could facilitate the development of new diagnostic tools for age-related learning difficulties.

### Limitations

One limitation of the present study might be the low task difficulty for young participants. Many young participants learned the sequence of stimuli already after the initial sequence presentation leading to no (or only few) UN, but only NL and K trials. This resulted in an unbalanced design with the majority of K trials and only a small portion of UN trials. However, we adjusted the task difficulty in a pilot study prior to data collection to the capabilities of the older participants, as the main goal of this study was to investigate the age-related learning trajectories. Another cause of poor recall in the older people could be that despite similar to young engagement of memory formation processes, not all of the learned material translated efficiently into solid sequence knowledge possibly due to poor learning strategy, or even if they are learning, they might make more mistakes in reporting that learning. P300 is not elicited or is reduced, when participants chose to ignore certain stimuli in a given sequence repetition in order to learn them in the next one. The older people may suffer interference effects during the recall itself while spending resources on using computer mouse which compromise their very recently made, still precarious memory trace and so they lose it. The younger people’s memory traces would be more resilient to having to use a mouse to report the locations, because they are more familiar with technology. Alternatively, some participants may have used subvocal verbal coding. Perhaps younger participants used it more than older participants, increasing group differences. If the locations were mentally numbered, like an eight-digit clock face, the supposedly visuo-spatial task would be transformed into a mixed visuo-spatial and auditory-verbal task, yielding better encoding and recall than relying only on visual information.

## Conclusion

Using a paradigm that provides the opportunity to investigate the incremental nature of learning, we gained novel insights into neural processes that contribute to age-related impeded memory. Our data demonstrate that the diminished learning capabilities in older participants were directly linked to longer P300 peak latencies, decreased P300 amplitudes, and more gradual modulations of the P300 and BP amplitudes over the course of learning reflecting more spread-out learning progress. The decreased P300 amplitude and longer P300 peak latencies may be interpreted as evidence of cognitive decline, which means that older participants require more time for stimulus evaluation and engage a lower level of attentional resources resulting in lower behavioral performance and less distinct confidence about the learned material. Although the older participants engage the memory formation process reflected by BP to a similar level, there is less time for it to translate the categorical stimulus location information into a solidified memory trace due to the slower and more variable timing of the stimulus identification process reflected in the P300 latency. We provide evidence that the P300 amplitude can predict learning success across sequence repetitions and identify fast and slow learners. The present report foregrounds the important role of the expectancy-driven P300 as potential biomarker for learning success, which may facilitate the development of preventive techniques for age-related impeded learning.

### Supplementary Information

Below is the link to the electronic supplementary material.

Supplementary file1 (DOCX 933 KB)

## Data Availability

All data, preprocessing, and analysis scripts used for the analyses are uploaded on OSF.io at https://osf.io/se8bf/. We agree to share our data, any digital study materials, and laboratory logs for all published results in this repository.

## Abbreviations

EEG: Electroencephalography
ERP: Event-related potential
BP: Broad positivity
mP300: Average P300
mBP: Average broad positivity
MMSE: Mini-mental state examination
AD: Alzheimer’s disease
ICA: Independent component analysis
ICC: Intraclass correlation coefficient
