# Supplementary material for: Neurophysiological markers of successful learning in healthy aging
Source: GeroScience. 2023 May 12;45(5):2873–96. doi: 10.1007/s11357-023-00811-8 (PMC10643715; doi:10.1007/s11357-023-00811-8)
Supplement: Supplementary file 1 — Supplementary file1 (DOCX 933 KB) [file 11357_2023_811_MOESM1_ESM.docx]

**Supplementary** **Material** - For publication as an online data supplement

To be submitted as Research Article to GeroScience

Neurophysiological markers of successful learning in healthy aging

AUTHORS:

Dawid Strzelczyk^1,2,3*^, Simon P. Kelly^4^, Nicolas Langer^1,2,3^

AFFILIATIONS:

1: Methods of Plasticity Research, Department of Psychology, University of Zurich, Zurich, Switzerland

2: University Research Priority Program (URPP) Dynamics of Healthy Aging, Zurich, Switzerland

3: Neuroscience Center Zurich (ZNZ), Zurich, Switzerland

4: School of Electrical and Electronic Engineering and UCD Centre for Biomedical Engineering, University College Dublin, Dublin, Ireland

E-mails:

dawid.strzelczyk@psychologie.uzh.ch, simon.kelly@ucd.ie, n.langer@psychologie.uzh.ch

ORCID:

Dawid Strzelczyk: 0000-0001-8344-1271

Simon P. Kelly: 0000-0001-9983-3595

Nicolas Langer: 0000-0002-6038-9471

CORRESPONDING AUTHOR (*):

Dawid Strzelczyk

Methods of Plasticity Research

Department of Psychology

University of Zurich

Andreasstrasse 15

CH-8050, Zürich, Switzerland

E-mail: dawid.strzelczyk@psychologie.uzh.ch

Tel.: +41 75 421 34 00

1. **Supplementary** **Figures**


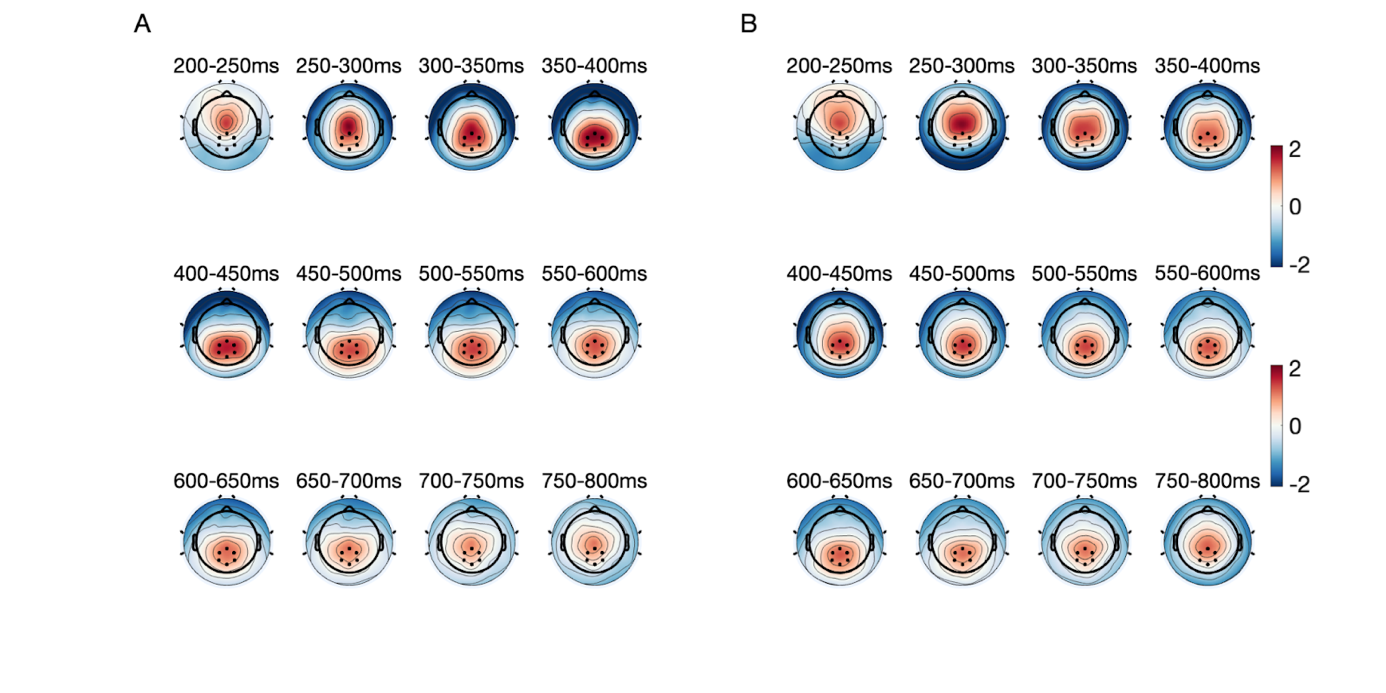


**Supplementary Fig. 1** Grand average scalp topographies plotted with a 50 ms step in young (A) and older (B) participants. The Grand average consists only of unknown and newly learned stimuli, because the known stimuli were not expected to elicit a clear P300 peak. The black dots indicate centro-parietal electrodes selected for computing ERPs and further statistical analysis.


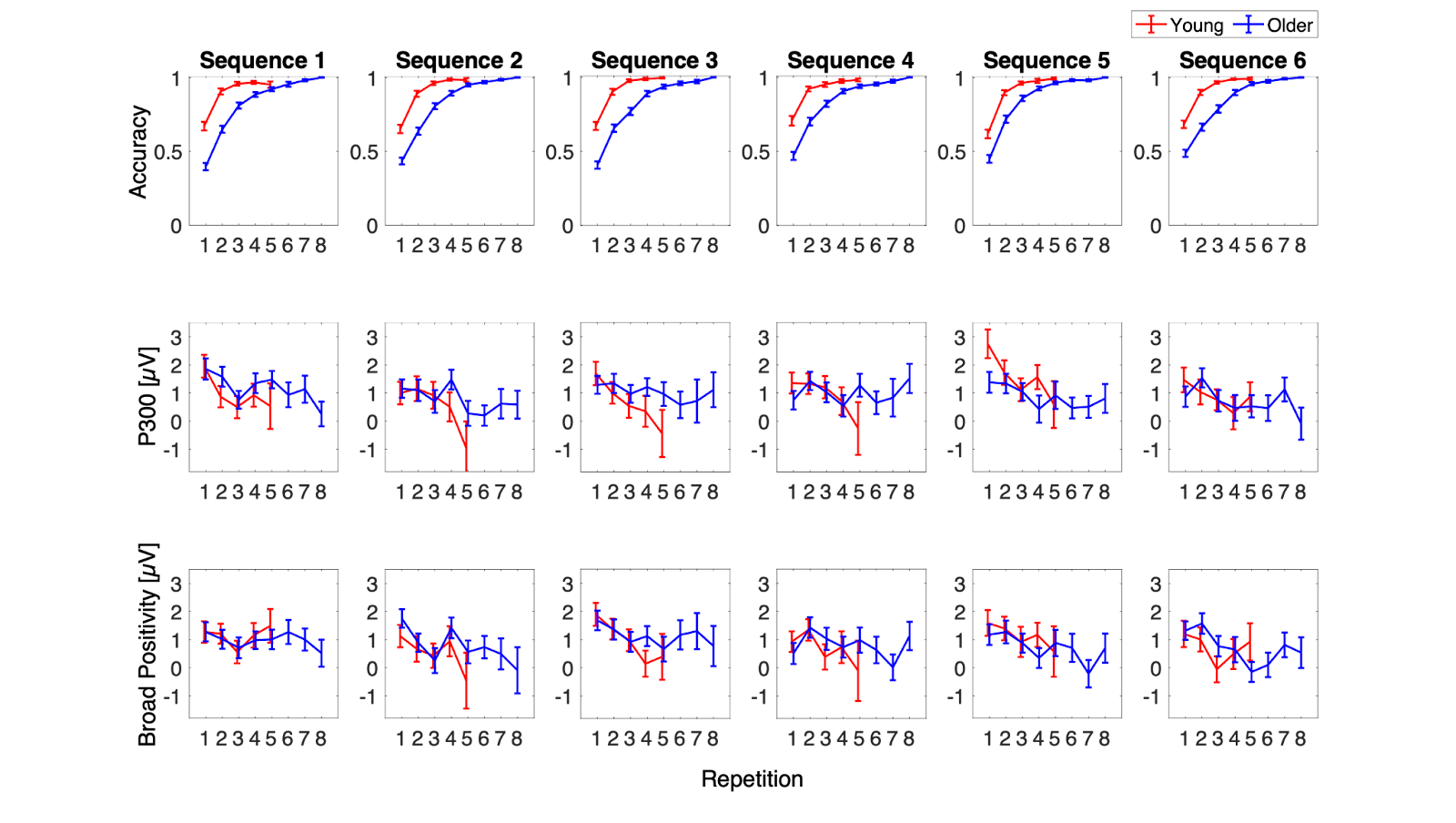


**Supplementary Fig. 2** Accuracy, P300 and broad positivity across all six stimulus sequences (three from the first and three from the second session) in young (red) and older (blue) participants. The accuracy increased monotonically in young and older individuals, however the young learned the sequence of stimuli faster than the older. Moreover, P300 and BP amplitudes decreased on average with increasing accuracy in both age groups. In young, only the first five sequence repetitions were plotted due to the low number of trials from the sixth repetition on. Error bars represent the standard error of the mean.


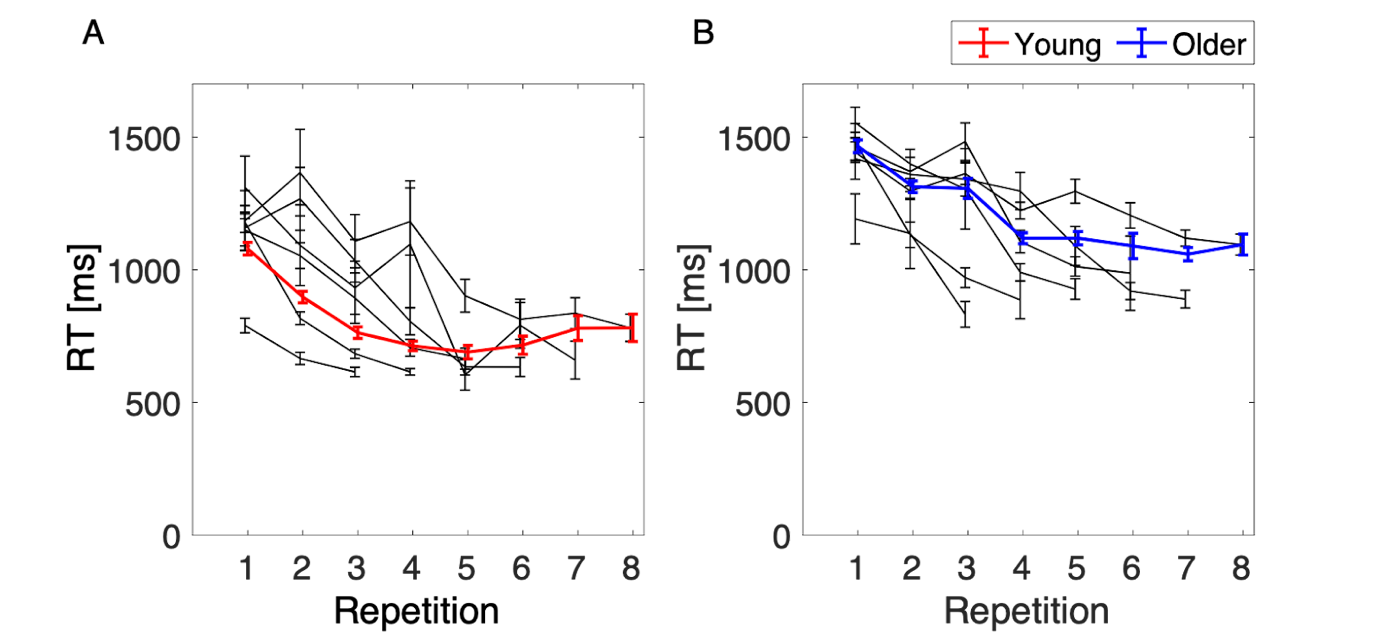


**Supplementary Fig. 3** Reaction times in young (A) and older (B) participants. Please note that the participants were not explicitly instructed to perform the task as fast as possible. Hence, the reaction times need to be interpreted with caution.

1. **Supplementary** **Tables**

*Accuracy*

The best-fit model for the accuracy included the fixed effect of repetition number, age group, and their interaction, and a fixed effect of session as well as the random effect of subject.

Accuracy ~ RepetitionNr * AgeGroup + Session + (1|Subject)

Supplementary Table 1. Effects of repetition number and age group on accuracy

| ***Variable*** | ***β*** | ***SE*** | ***CI*** | ***t-value*** | ***p-value*** |
| --- | --- | --- | --- | --- | --- |
| Intercept | 0.68 | 0.01 | 0.66 – 0.70 | 59.74 | 2.5e-249*** |
| RepetitionNr | 0.07 | 0.002 | 0.07 – 0.08 | 30.56 | 1.7e-191*** |
| AgeGroup | -0.21 | 0.02 | -0.24 – -0.18 | -14.40 | 2.6e-39*** |
| RepetitionNr *AgeGroup | 0.01 | 0.003 | -0.03 – -0.01 | 5.06 | 4.4e-7*** |
| Session 2 | 0.02 | 0.005 | 0.01 – 0.03 | 4.28 | 1.9e-5*** |
| **Variance components** | **SD** | **Goodness of fit** | |  |  |
| Subject | 0.08 | Log likelihood | | 1565.17 |  |
| Residual | 0.18 |  |  |  |  |

*Note. Intercept represents the first repetition of young participants. RepetitionNr = Repetition number. β = unstandardized regression coefficient. SE = Standard error. CI = Confidence interval. SD = Standard deviation.*

**p < 0.05. **p < 0.01. ***p < 0.001.*

*Learning rate*

The best-fit model for the learning rate included only fixed effects: the repetition number, age group and their interaction. The step function eliminated all random effects suggesting no substantial variance in the learning rate between participants or sequences. Therefore, subsequent results represent the coefficients of a simple linear model.

Learning rate ~ RepetitionNr * AgeGroup

Supplementary Table 2. Effects of repetition number and age group on learning rate

| ***Variable*** | ***β*** | ***SE*** | ***CI*** | ***t-value*** | ***p-value*** |
| --- | --- | --- | --- | --- | --- |
| Intercept | 0.57 | 0.01 | 0.55 – 0.59 | 59.93 | 1e-314*** |
| RepetitionNr | -0.11 | 0.003 | -0.12 – -0.11 | -39.35 | 1.5e-303*** |
| AgeGroup | -0.22 | 0.01 | -0.24 – -0.19 | -17.75 | 7.2e-69*** |
| RepetitionNr *AgeGroup | 0.07 | 0.003 | 0.07 – 0.08 | 22.21 | 2.4e-105*** |
| **Variance components** | **SD** | **Goodness of fit** | |  |  |
| Residual | 0.23 | Log likelihood | | 283.51 |  |

*Note. Intercept represents the first repetition of young participants. RepetitionNr = Repetition number. β = unstandardized regression coefficient. SE = Standard error. CI = Confidence interval. SD = Standard deviation.*

**p < 0.05. **p < 0.01. ***p < 0.001.*

*P300 over sequence repetitions*

The best-fit model for average P300 amplitude included the fixed effects of repetition number, age group, baseline, interaction of repetition number and age group, interaction of repetition number and baseline. The random part consisted of the subject and sequence number.

mP300 ~ RepetitionNr * AgeGroup + RepetitionNr * Baseline + (1|Subject) + (1|SequenceNr)

Supplementary Table 3. Effects of repetition number and age group on P300 amplitude

| ***Variable*** | ***β*** | ***SE*** | ***CI*** | ***t-value*** | ***p-value*** |
| --- | --- | --- | --- | --- | --- |
| Intercept | 1.37 | 0.07 | 1.24 – 1.51 | 21.01 | 8.6e-51*** |
| RepetitionNr | -0.18 | 0.01 | -0.21 – -0.15 | -13.53 | 3.7e-41*** |
| AgeGroup | -0.26 | 0.08 | -0.40 – -0.10 | -3.24 | 0.001** |
| RepetitionNr *AgeGroup | 0.11 | 0.02 | 0.08 – 0.14 | 7.47 | 8.6e-14*** |
| Baseline | -0.29 | 0.03 | -0.35 – -0.24 | -10.6 | 5e-26*** |
| RepetitionNr*Baseline | 0.03 | 0.007 | 0.01 – 0.04 | 4.14 | 3.6e-5*** |
| **Variance components** | **SD** | **Goodness of fit** | |  |  |
| Subject | 0.42 | Log likelihood | | -9292.25 |  |
| SequenceNr | 0.05 |  | |  |  |
| Residual | 0.99 |  | |  |  |

*Note. Intercept represents the first repetition of young participants. RepetitionNr = Repetition number. SequenceNr = Sequence number. β = unstandardized regression coefficient. SE = Standard error. CI = Confidence interval. SD = Standard deviation.*

**p < 0.05. **p < 0.01. ***p < 0.001.*

*Broad Positivity over sequence repetitions*

Subsequently, we identified the best-fit model for the average broad positivity amplitude across sequence repetitions.

mBP ~ RepetitionNr * AgeGroup + Baseline + Gender + (1|Subject)

Supplementary Table 4. Effects of repetition number and age group on BP amplitude

| ***Variable*** | ***β*** | ***SE*** | ***CI*** | ***t-value*** | ***p-value*** |
| --- | --- | --- | --- | --- | --- |
| Intercept | 0.89 | 0.07 | 0.78 – 1.01 | 14.69 | 1.2e-41*** |
| RepetitionNr | -0.11 | 0.01 | -0.14 – -0.08 | -8.24 | 5e-16*** |
| AgeGroup | -0.13 | 0.08 | -0.27 – 0.01 | -1.93 | 0.054 |
| RepetitionNr *AgeGroup | 0.06 | 0.02 | 0.01 – 0.08 | 3.59 | 3.3e-4*** |
| Gender Female | 0.1 | 0.05 | 0.01 – 0.20 | 2.01 | 0.044* |
| Baseline | -0.13 | 0.01 | -0.15 – -0.10 | -9.19 | 5.1e-20*** |
| **Variance components** | **SD** | **Goodness of fit** | |  |  |
| Subject | 0.30 | Log likelihood | | -9440.81 |  |
| Residual | 1.06 |  | |  |  |

*Note. Intercept represents the first repetition of young participants. RepetitionNr = Repetition number. β = unstandardized regression coefficient. SE = Standard error. CI = Confidence interval. SD = Standard deviation.*

**p < 0.05. **p < 0.01. ***p < 0.001.*

*Accuracy and P300*

Expectancy driven P300 was hypothesized to decrease, as the sequence knowledge strengthened. Thus, we tested whether the P300 amplitude could predict the accuracy across sequence repetitions. First, we identified the best-fit model for predicting the accuracy. The model included fixed effects of average P300, age group, session and average baseline, as well as the interaction of average P300 and baseline. The random effects included only the subject.

Accuracy ~ mP300 + AgeGroup + Baseline + Session + mP300 * Baseline + (1|Subject)

Supplementary Table 5. Effects of P300 on accuracy

| ***Variable*** | ***β*** | ***SE*** | ***CI*** | ***t-value*** | ***p-value*** |
| --- | --- | --- | --- | --- | --- |
| Intercept | 0.92 | 0.01 | 0.90 – 0.93 | 125.96 | 1e-314*** |
| mP300 | -0.04 | 0.003 | -0.04 – -0.03 | -12.68 | 2.2e-36*** |
| AgeGroup | -0.09 | 0.01 | -0.10 – -0.07 | -11.18 | 2.5e-23*** |
| Session 2 | 0.01 | 0.006 | 0.00 – 0.02 | 2.11 | 0.035* |
| Baseline | -0.002 | 0.004 | -0.01 – 0.01 | -0.52 | 0.601 |
| mP300*Baseline | -0.004 | 0.002 | -0.01 – -0.00 | -2.6 | 0.009** |
| **Variance components** | **SD** | **Goodness of fit** | |  |  |
| Subject | 0.03 | Log likelihood | | -35.69 |  |
| Residual | 0.26 |  | |  |  |

*Note. Intercept represents the young participants. mP300 = Mean P300. β = unstandardized regression coefficient. SE = Standard error. CI = Confidence interval. SD = Standard deviation.*

**p < 0.05. **p < 0.01. ***p < 0.001.*

*Learning rate and broad positivity*

BP, the signal thought to reflect active memory trace formation, was expected to be maximal for stimuli being actively committed to memory. Thus, we tested whether the learning rate, which represents the proportion of newly learned stimuli in a given repetition, could be predicted by the average BP amplitude across sequence repetitions. First, we identified the best-fit model for predicting the learning rate. The model included fixed effects of average BP, age group, average baseline, as well as the interaction of average BP and age group, and interaction of age group and baseline.

Learning rate ~ mBP * AgeGroup + AgeGroup * Baseline

Supplementary Table 6. Effects of BP on learning rate

| ***Variable*** | ***β*** | ***SE*** | ***CI*** | ***t-value*** | ***p-value*** |
| --- | --- | --- | --- | --- | --- |
| Intercept | 0.20 | 0.01 | 0.19 – 0.21 | 30.771 | 2.2e-193*** |
| mBP | 0.05 | 0.01 | 0.04 – 0.06 | 10.42 | 3.1e-25*** |
| AgeGroup | 0.004 | 0.01 | -0.01 – 0.02 | 0.49 | 0.621 |
| mBP*AgeGroup | -0.04 | 0.01 | -0.05 – -0.03 | -6.24 | 4.5e-10*** |
| Baseline | -0.02 | 0.01 | -0.03 – -0.01 | -4.54 | 5.7e-6*** |
| AgeGroup*Baseline | 0.03 | 0.01 | 0.02 – 0.05 | 4.76 | 1.9e-6*** |
| **Variance components** | **SD** | **Goodness of fit** | |  |  |
| Residual | 0.26 | Log likelihood | | -515.91 |  |

*Note. Intercept represents the young participants. mBP = Mean broad positivity. β = unstandardized regression coefficient. SE = Standard error. CI = Confidence interval. SD = Standard deviation.*

**p < 0.05. **p < 0.01. ***p < 0.001.*

*Predicting learning success across participants*

The best-fit model included the fixed main effects of P300 decrease and age group, interaction of age group and P300 decrease, as well as random effects of subject and sequence number.

numberRepetitions ~ P300 decrease * AgeGroup + (1|Subject) + (1|SequenceNr)

Supplementary Table 7. Learning success prediction across participants

| ***Variable*** | ***β*** | ***SE*** | ***CI*** | ***t-value*** | ***p-value*** |
| --- | --- | --- | --- | --- | --- |
| Intercept | 4.52 | 0.11 | 4.30 – 4.75 | 39.26 | 1.1e-46*** |
| P300 decrease | 0.08 | 0.02 | 0.03 – 0.12 | 3.29 | 0.001** |
| AgeGroup | 1.91 | 0.14 | 1.62 – 2.19 | 13.40 | 4e-30*** |
| P300 decrease * AgeGroup | -0.08 | 0.03 | -0.15 – -0.01 | -2.29 | 0.022* |
| **Variance components** | **SD** | **Goodness of fit** | |  |  |
| Subject | 0.88 | Log likelihood | | -1959.50 |  |
| SequenceNr | 0.11 |  | |  |  |
| Residual | 1.31 |  | |  |  |

*Note. Intercept represents the young participants. SequenceNr = Sequence number. β = unstandardized regression coefficient. SE = Standard error. CI = Confidence interval. SD = Standard deviation.*

**p < 0.05. **p < 0.01. ***p < 0.001.*

*P300 across learning states*

The best-fit model for P300 amplitude included the fixed effect of category, age group, baseline and their 3-way interaction, and random effects of subject, stimulus number, repetition number and sequence number.

P300 ~ Category * AgeGroup * Baseline + (1|Subject) + (1|StimulusNr) + (1|RepetitionNr) + (1|SequenceNr)

Supplementary Table 8. Effects of age group and learning categories on P300 amplitude

| ***Variable*** | ***β*** | ***SE*** | ***CI*** | ***t-value*** | ***p-value*** |
| --- | --- | --- | --- | --- | --- |
| Intercept | 1.15 | 0.10 | 0.96 – 1.34 | 11.23 | 6.5e-13*** |
| Category K | -0.62 | 0.05 | -0.72 – -0.53 | -12.96 | 2.6e-38*** |
| Category UN | -0.12 | 0.06 | -0.24 – 0.00 | -1.91 | 0.056 |
| AgeGroup | -0.21 | 0.08 | -0.36 – -0.06 | -2.74 | 0.006** |
| Category K*AgeGroup | 0.43 | 0.06 | 0.32 – 0.55 | 7.47 | 8.5e-14*** |
| Category UN*AgeGroup | 0.07 | 0.07 | -0.07 – 0.22 | 0.96 | 0.338 |
| Baseline | -0.28 | 0.02 | -0.31 – -0.25 | -19.25 | 3.2e-82*** |
| Category K*Baseline | 0.08 | 0.02 | 0.04 – 0.11 | 4.51 | 6.6e-06*** |
| Category UN*Baseline | 0.11 | 0.02 | 0.06 – 0.15 | 4.52 | 6.1e-06*** |
| AgeGroup*Baseline | 0.12 | 0.02 | 0.08 – 0.16 | 6.18 | 6.4e-10*** |
| Category K * AgeGroup * Baseline | -0.07 | 0.02 | -0.11 – -0.02 | -2.90 | 0.004** |
| Category UN * AgeGroup * Baseline | -0.07 | 0.03 | -0.13 – -0.01 | -2.32 | 0.02* |
| **Variance components** | **SD** | **Goodness of fit** | |  |  |
| Subject | 0.42 | Log likelihood | | -104793.5 |  |
| StimulusNr | 0.14 |  | |  |  |
| RepetitionNr | 0.15 |  | |  |  |
| SequenceNr | 0.05 |  | |  |  |
| Residual | 2.36 |  | |  |  |

*Note. Intercept represents the newly learned category of young participants. UN = Unknown. NL = Newly learned. K = Known. StimulusNr = Stimulus number. RepetitionNr = Repetition number. SequenceNr = Sequence number. β = unstandardized regression coefficient. SE = Standard error. CI = Confidence interval. SD = Standard deviation.*

**p < 0.05. **p < 0.01. ***p < 0.001.*

*Broad Positivity across learning states*

The best-fit model included fixed effects of category, age group, baseline, gender and an interaction of category, age group and baseline and the random effect of subject, stimulus number and repetition number.

BP ~ Category * AgeGroup * Baseline + Session + (1|Subject) + (1|StimulusNr) + (1|RepetitionNr)

Supplementary Table 9. Effects of age group and learning categories on BP amplitude

| ***Variable*** | ***β*** | ***SE*** | ***CI*** | ***t-value*** | ***p-value*** |
| --- | --- | --- | --- | --- | --- |
| Intercept | 0.90 | 0.07 | 0.77 – 1.03 | 13.42 | 3.3e-19*** |
| Category K | -0.43 | 0.05 | -0.53 – -0.33 | -8.63 | 2e-17*** |
| Category UN | -0.11 | 0.07 | -0.24 – 0.02 | 1.67 | 0.095 |
| AgeGroup | -0.15 | 0.07 | -0.28 – -0.02 | -2.31 | 0.021* |
| Category K*AgeGroup | 0.16 | 0.06 | 0.04 – 0.29 | 2.65 | 0.008** |
| Category UN*AgeGroup | 0.10 | 0.08 | -0.06 – 0.25 | 1.34 | 0.216 |
| Session 2 | -0.05 | 0.02 | -0.10 – -0.00 | -2.05 | 0.041* |
| Baseline | -0.16 | 0.02 | -0.19 – -0.12 | 10.05 | 1e-23*** |
| Category K*Baseline | -0.01 | 0.02 | -0.05 – 0.02 | -0.68 | 0.495 |
| Category UN*Baseline | 0.04 | 0.03 | -0.01 – 0.08 | 1.40 | 0.161 |
| AgeGroup*Baseline | 0.01 | 0.02 | -0.03 – 0.06 | 0.70 | 0.485 |
| Category K * AgeGroup * Baseline | 0.04 | 0.02 | -0.01 – 0.09 | 1.56 | 0.119 |
| Category UN * AgeGroup * Baseline | -0.04 | 0.03 | -0.10 – -0.02 | -1.20 | 0.230 |
| **Variance components** | **SD** | **Goodness of fit** | |  |  |
| Subject | 0.30 | Log likelihood | | -107976.7 |  |
| StimulusNr | 0.10 |  | |  |  |
| RepetitionNr | 0.06 |  | |  |  |
| Residual | 2.53 |  | |  |  |

*Note. Intercept represents the newly learned category of young participants. UN = Unknown. NL = Newly learned. K = Known. StimulusNr = Stimulus number. RepetitionNr = Repetition number. β = unstandardized regression coefficient. SE = Standard error. CI = Confidence interval. SD = Standard deviation.*

**p < 0.05. **p < 0.01. ***p < 0.001.*
